# Structure of the MIS12 Complex and Molecular Basis of Its Interaction with CENP-C at Human Kinetochores

**DOI:** 10.1016/j.cell.2016.10.005

**Published:** 2016-11-03

**Authors:** Arsen Petrovic, Jenny Keller, Yahui Liu, Katharina Overlack, Juliane John, Yoana N. Dimitrova, Simon Jenni, Suzan van Gerwen, Patricia Stege, Sabine Wohlgemuth, Pascaline Rombaut, Franz Herzog, Stephen C. Harrison, Ingrid R. Vetter, Andrea Musacchio

**Affiliations:** 1 Department of Mechanistic Cell Biology, Max Planck Institute of Molecular Physiology, Otto-Hahn-Straße 11, 44227 Dortmund, Germany; 2 Department of Biological Chemistry and Molecular Pharmacology, Harvard Medical School, 250 Longwood Avenue, Boston, MA 02115; 3 Gene Center Munich, Ludwig-Maximilians-Universität München, Feodor-Lynen-Str. 25, 81377 Munich, Germany; 4 Department of Biological Chemistry and Molecular Pharmacology, Howard Hughes Medical Institute, Harvard Medical School, 250 Longwood Avenue, Boston, MA 02115; 5 Faculty of Biology, Centre for Medical Biotechnology, University Duisburg-Essen, Universitätsstrasse, 45141 Essen, Germany

**Keywords:** CENP-C, KMN network, Mis12, PMF1, DSN1, NSL1, MIND, kinetochore, centromere, CCAN

## Abstract

Kinetochores, multisubunit protein assemblies, connect chromosomes to spindle microtubules to promote chromosome segregation. The 10-subunit KMN assembly (comprising KNL1, MIS12, and NDC80 complexes, designated KNL1C, MIS12C, and NDC80C) binds microtubules and regulates mitotic checkpoint function through NDC80C and KNL1C, respectively. MIS12C, on the other hand, connects the KMN to the chromosome-proximal domain of the kinetochore through a direct interaction with CENP-C. The structural basis for this crucial bridging function of MIS12C is unknown. Here, we report crystal structures of human MIS12C associated with a fragment of CENP-C and unveil the role of Aurora B kinase in the regulation of this interaction. The structure of MIS12:CENP-C complements previously determined high-resolution structures of functional regions of NDC80C and KNL1C and allows us to build a near-complete structural model of the KMN assembly. Our work illuminates the structural organization of essential chromosome segregation machinery that is conserved in most eukaryotes.

## Highlights

• We report a crystal structure of human MIS12 complex, a crucial kinetochore component
• The structure reveals how the MIS12 complex binds its kinetochore receptor CENP-C
• We dissect how Aurora B kinase promotes the MIS12:CENP-C interaction
• A combination of diverse structural methods reveals outer kinetochore organization

## Graphical Abstract

**Figure:**
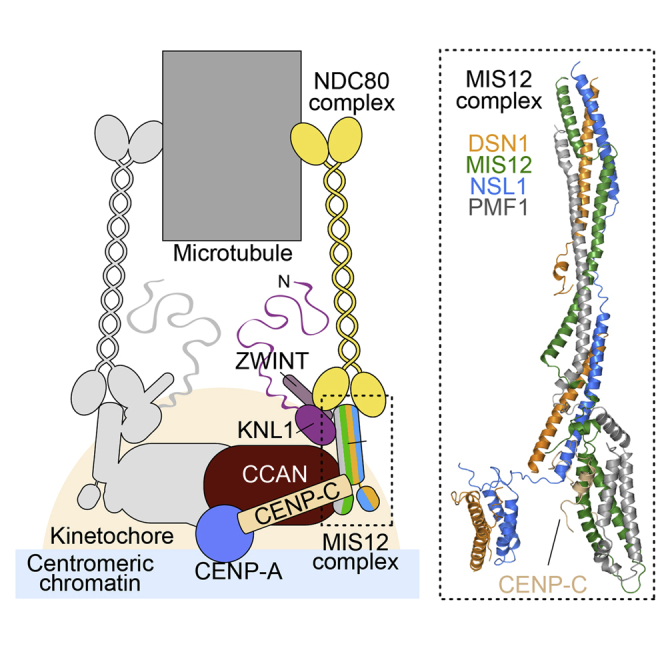

## Teaser

Structural analyses show how a human kinetochore subcomplex serves as an adaptor between centromeric nucleosomes and outer kinetochore components.

## Introduction

In eukaryotes, chromosomes are replicated during the S-phase (synthesis) of the cell cycle and then segregated from a mother cell to its two daughters during M-phase (mitosis). Accurate segregation of the sister chromatids (replicated chromosomes) requires their interplay with the mitotic spindle, which self-assembles through the combined action of microtubules, microtubule motors, and microtubule-binding proteins (Foley and Kapoor, 2013).

**Figure S1:**
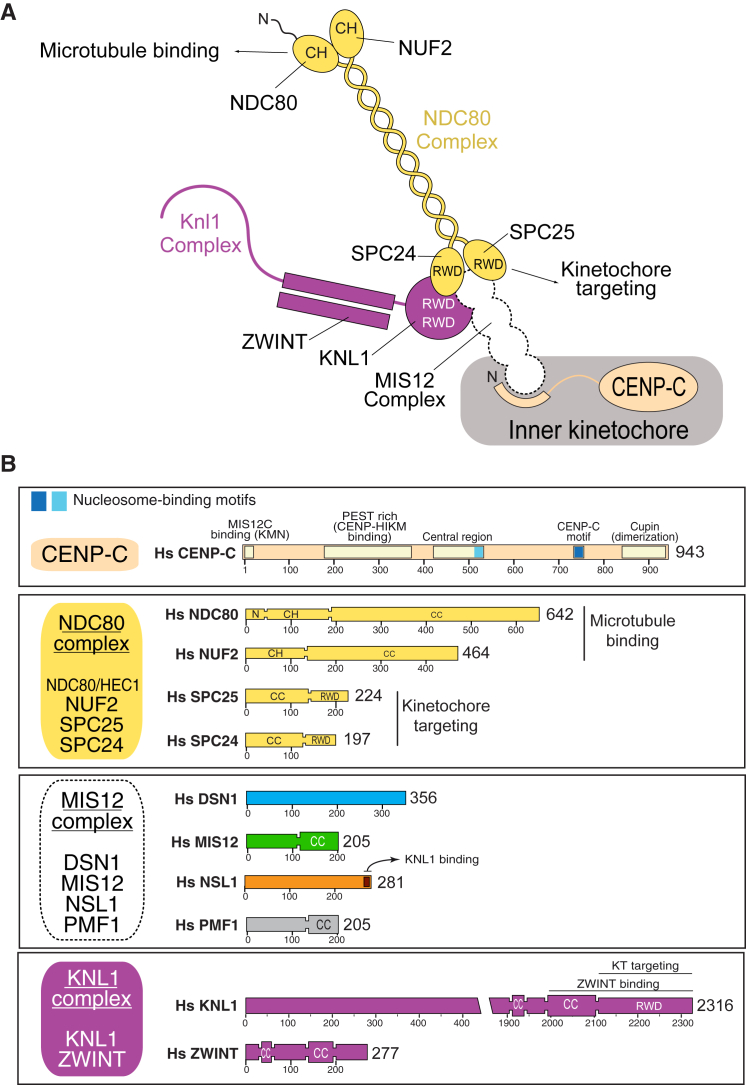
Organization of the KMN Network, Related to Figure 1 (A) Drawing of the KMN network and its interaction with CCAN. CH, Calponin-homology domain; RWD, RING finger, WD repeat, DEAD-like helicases. (B) Schematic representation of the organization of subunits of the KMN network and main functional domains; CC, coiled-coil; N, N-terminal tail (involved in microtubule binding); PEST, proline-glutamic-serine-threonine. The CENP-C motif and the central region of CENP-C contain conserved nucleosome binding motifs.

Kinetochores mediate the physical interaction of chromosomes with the mitotic spindle and ensure that sister chromatids bi-orient, i.e., that they bind to opposite poles of the mitotic spindle, a condition required for accurate chromosome segregation (Foley and Kapoor, 2013, London and Biggins, 2014). Kinetochores, which are largely conserved in eukaryotes, contain ∼26 core subunits and an array of regulatory subunits (Pesenti et al., 2016). The core subunits have been classified into two distinct assemblies, designated constitutive centromere-associated network (CCAN) and Knl1 complex; Mis12 complex; Ndc80 complex (KMN), the former loosely associated with chromatin components and the latter, with spindle microtubules (McKinley and Cheeseman, 2016, Pesenti et al., 2016; Figure S1A). The 16 or more CCAN subunits fall into several discrete complexes (McKinley and Cheeseman, 2016, Pesenti et al., 2016). Of these, CENP-C and the CENP-LN complex interact directly with the centromere-specific histone H3 variant CENP-A, considered the linchpin of kinetochore assembly (Carroll et al., 2009, Carroll et al., 2010, Kato et al., 2013, Weir et al., 2016).

The 10-subunit KMN assembly consists of three complexes, the 4-subunit MIS12 complex (MIS12C), the 2-subunit KNL1 complex (KNL1C), and the 4-subunit NDC80 complex (NDC80C) (Bharadwaj et al., 2004, Cheeseman et al., 2004, De Wulf et al., 2003, Desai et al., 2003, Kline et al., 2006, McCleland et al., 2003, Nekrasov et al., 2003, Obuse et al., 2004, Pinsky et al., 2003, Westermann et al., 2003, Wigge and Kilmartin, 2001). Human MIS12C (also known as MIND complex or Mtw1 complex in *Saccharomyces cerevisiae*) contains the MIS12, PMF1, NSL1, and DSN1 subunits (Figure S1B; synonyms are reported in Table S1A). KNL1C (known as Spc105 complex in *S. cerevisiae*) contains the KNL1 and ZWINT subunits. Finally, NDC80C contains the NDC80, NUF2, SPC24, and SPC25 subunits.

The three KMN sub-complexes are functionally distinct. NDC80C is a long coiled-coil with globular domains at both ends and an end-to-end length of ∼55 nm (Ciferri et al., 2005, Wei et al., 2005). Its two sub-complexes, NDC80:NUF2 and SPC24:SPC25, are responsible for microtubule binding and kinetochore localization, respectively (Cheeseman et al., 2006, Ciferri et al., 2008, DeLuca et al., 2005, DeLuca et al., 2006, Malvezzi et al., 2013, Petrovic et al., 2010, Wei et al., 2006, Wei et al., 2007). KNL1, the largest core kinetochore protein in humans (2,316 residues), is implicated in mitotic checkpoint control (reviewed in London and Biggins, 2014). Finally, MIS12C is a binding hub that connects the other two KMN complexes to CCAN through an interaction with CENP-C (Gascoigne et al., 2011, Hori et al., 2013, Hornung et al., 2011, Hornung et al., 2014, Liu et al., 2016, Maskell et al., 2010, Petrovic et al., 2010, Przewloka et al., 2011, Screpanti et al., 2011). Aurora B phosphorylation at Ser100 and Ser109 of human DSN1 enhances the CENP-C:MIS12C interaction (Kim and Yu, 2015, Rago et al., 2015, Welburn et al., 2010, Yang et al., 2008), and the pathway is conserved in *S. cerevisiae* (Akiyoshi et al., 2013).

In recent years, there has been substantial progress in the biochemical and structural characterization of KMN network components. Negative-stain electron microscopy (EM) demonstrated that MIS12C is elongated, with a long axis of ≈20 nm and one end thinner than the other (Hornung et al., 2011, Maskell et al., 2010, Petrovic et al., 2010). EM analysis of larger KMN reconstitutions, including a low-resolution 3D negative stain EM reconstruction of MIS12C bound to a segment of KNL1 and to an engineered chimeric construct of NDC80C called NDC80C^Bonsai^ (Ciferri et al., 2008), suggested that MIS12C interacts with NDC80C and KNL1C near the thinner end; that MIS12C and NDC80C bind “in series” to form an ≈80 nm structure; and that KNL1 departs from the axis of the NDC80:MIS12 rod at an ≈65° angle (Petrovic et al., 2014, Screpanti et al., 2011).

While the previous work began to illustrate the structural organization of MIS12C, a high-resolution structure of this complex has been missing. We have now overcome this limitation by determining the crystal structure of the human MIS12C in complex with the N-terminal region of CENP-C, previously implicated in MIS12C binding (Przewloka et al., 2011, Screpanti et al., 2011). The resulting model reveals the overall molecular organization of the MIS12C and the details of its interaction with CENP-C. We clarify how Aurora B phosphorylation of DSN1 enhances the CENP-C:MIS12C interaction. Finally, we build a near complete structural model of the KMN assembly and discuss its implications for microtubule binding and CCAN regulation.

## Results

### Crystal Structure of Human MIS12C

**Figure S2:**
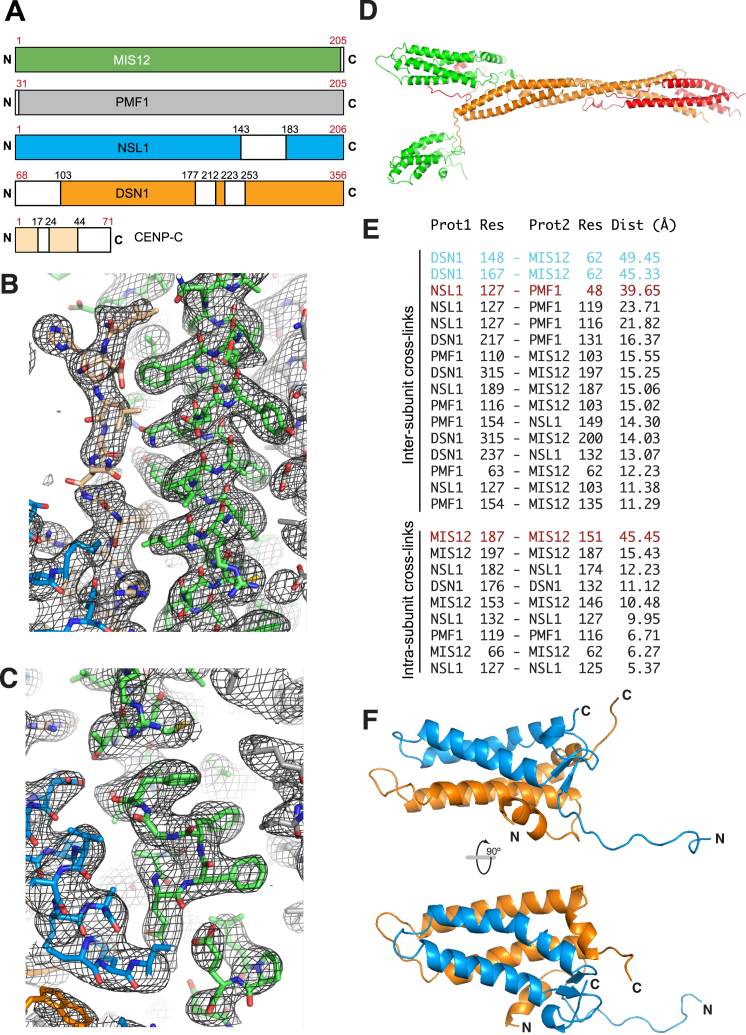
Electron Density Maps, Related to Figure 1 (A) Results of limited proteolysis experiment with Chymotrypsin at protease:substrate ratios similar to that used in crystallization experiment. The red numbers indicate the boundaries of the MIS12^Nano^ construct (see Table S1). Black numbers indicate boundaries of protease-trimmed segments, shown in white. Some of the trimmed segments, however, were clearly visible in the crystal’s electron density, suggesting that proteolysis is less efficient in the crystallization buffer. See STAR Methods section ‘Crystal structure determination’ for additional details. (B and C) Snapshots of electron density for the MIS12C full length model. The shown map is an omit map, obtained with Phenix (Adams et al., 2010), contoured at 1 sigma (panel A) or at 1.5 sigma (panel B). In B, three Phe residues in the Phe-Phe-Gly-Phe motif of MIS12 are shown. The color scheme is the same already used in Figure 1. (D) Cartoon model of the MIS12C colored according to confidence of model building, from highest confidence (green) to medium (orange) to lowest (red). (E) Intra MIS12C cross-links, extracted from Tables S2 and S3, were mapped onto the final model of the MIS12C, and distances between Cα atoms of cross-linked lysines were tabulated. Cross-links were shown in black if the calculated distance between Cα atoms was compatible with formation of a cross-link, i.e., if it was less or equal to the combined length of the cross-linker (11.4 Å) and of two extended lysine side chains (∼6.3 Å, and therefore ∼24 Å in total). Two cross-links indicated in blue may reflect large-scale relative movements of Head2 and Head1 (possibly reflecting the Aurora B-regulated intra-molecular interaction). Only two cross-links (shown in brown) were inconsistent with the model, but they could reflect temporary fluctuations in the structure of the MIS12C. (F) Cartoon model of the Head2 structure in two orientations (Nsl1, blue; Dsn1, orange).

From the results of limited proteolysis experiments (Petrovic et al., 2014), we engineered a deletion construct of the MIS12C in which three of the four subunits carried N- or C-terminal deletions (Table S1B). The resulting construct, referred to as MIS12C^Nano^, did not crystallize, but addition of residues 1–71 of human CENP-C (CENP-C^1–71^), coupled with in situ proteolysis (Dong et al., 2007; Figure S2A; STAR Methods), led to crystals. We collected X-ray diffraction data to minimum Bragg spacing of 3.5 Å from a native crystal and obtained initial phases from a tantalum bromide (Ta_6_Br_12_^2+^) derivative in a different crystal form (Table 1). We used density modification methods and multi-crystal averaging to extend phases and obtain electron density maps with clear protein-solvent boundaries (Figures S2B and S2C). Model building was carried out as discussed in the STAR Methods. Collectively, the crystallographic work addresses the majority of the sequence of the human MIS12C and provides an excellent account of distance restraints derived from cross-linking and mass spectrometry analyses (Herzog et al., 2012), as summarized in Figures S2D and S2E (see also Tables S2 and S3).

**Figure 1:**
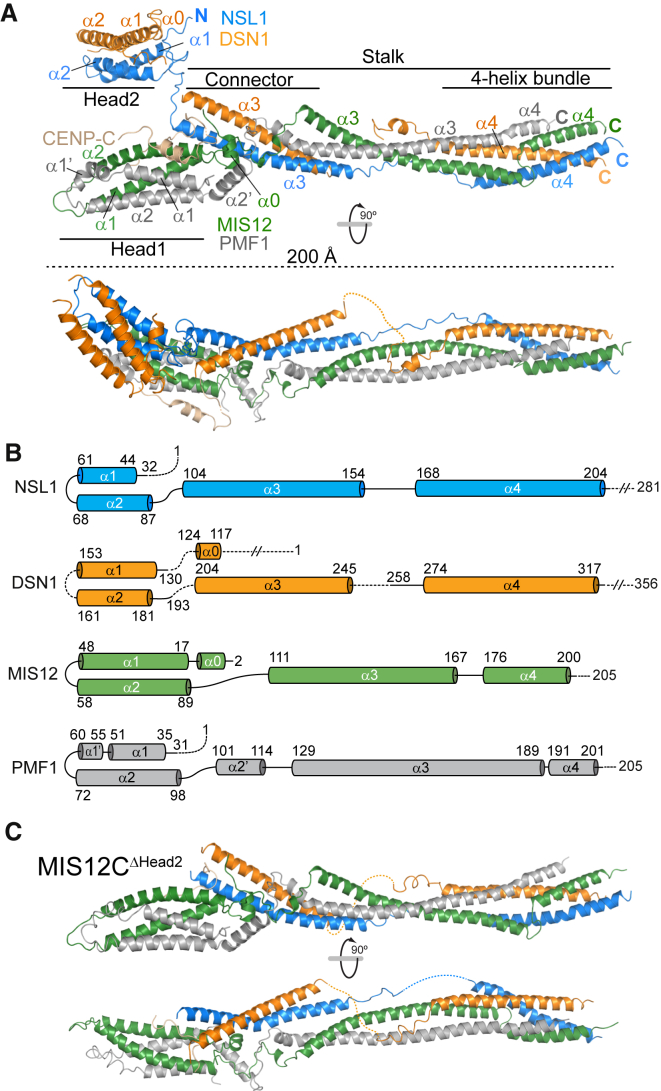
Structure of MIS12C:CENP-C (A) Cartoon diagrams of the MIS12C^Nano^:CENP-C^1–71^ complex (Table S1B) in two orientations. The main structural domains discussed in the text are indicated. The molecular models in this and subsequent figures were generated with PyMOL. The final model contains residues 2–200 of MIS12, 31–203 of PMF1, 32–204 of NSL1, 116–155, 159–193, 203–245, and 258–317 of DSN1. (B) Topology diagrams of the MIS12C subunits. (C) Cartoon diagrams of the MIS12C^ΔHead2^:CENP-C^1–71^ complex. The coloring scheme of subunits is as for the MIS12^Nano^ complex shown in (A). See also Figures S1 and S2.

The MIS12C is an extended rod, with a long axis of ∼200 Å (1 Å = 0.1 nm), in line with the previous low-resolution EM analyses (Figure 1A). The four MIS12C subunits have similar topologies (Figure 1B) and span the entire length of the complex in the same orientation, so that all N and C termini cluster at opposite ends. The MIS12C subunits form two distinct subcomplexes, MIS12:PMF1 and DSN1:NSL1. The buried surface areas (Collaborative Computational Project, Number 4, 1994) for the MIS12:PMF1 and DSN1:NSL1 pairs (4,209 and 3,575 Å^2^) are much larger than those of the four other interfaces, which range between 465 and 1,905 Å^2^. Both subcomplexes start with N-terminal helical hairpins that interact in four-helix bundles (defined here as “Head1” and “Head2” domains for the MIS12:PMF1 and DSN1:NSL1 subcomplexes, respectively) and progress into a “stalk” region (Figures 1A and 1B). The α3 helices of DSN1 and NSL1 pair in a helical segment (essentially a short segment of coiled-coil), with α3 of NSL1 kinking at the well-conserved Pro130^Nsl1^. The helical connector joins Head2 to the stalk, but Head2 itself is less restrained, as its linkage to the helical connector is extended and apparently flexible. The connection of Head1 to the stalk appears to be more fixed. The short α0 helix of the MIS12 subunit is largely buried at an interface of Head1 with the helical connector of DSN1:NSL1. The core of the stalk consists of the long and tightly interacting α3 helices of MIS12 and PMF1. The DSN1 and NSL1 chains in this region are extended and partly disordered and pack against the outer surface of the α3 helices of MIS12 and PMF1. It is possible that this region of MIS12C undergoes tension-dependent conformational rearrangements when NDC80C binds to microtubules, analogous to those recently proposed for the NDC80C (Suzuki et al., 2016). The DSN1 and NSL1 chains resume helical conformation to establish a four-helix bundle that engages the visible C termini of all four subunits. As explained in the context of Figure 5, this region is an interaction node where MIS12C binds with the NDC80C and KNL1C.

In *Drosophila melanogaster*, no homolog of the DSN1 subunit has been identified, but biochemical reconstitution with the remaining three subunits resulted in a very stable MIS12C, unlike reconstitutions in which either of the homologs of the MIS12 or PMF1 subunits had been omitted, resulting in unstable complexes (Liu et al., 2016, Richter et al., 2016). These recent observations confirm that MIS12 and PMF1 form the “backbone” of the MIS12C and suggest that NSL1 can adapt and retain significant stability even in the absence of DSN1.

We speculated that the detachment of Head2 from the core of the MIS12C might, at least in part, explain our difficulties in obtaining well-ordered crystals of MIS12C. We therefore generated a deletion construct, referred to as MIS12C^ΔHead2^ (i.e., lacking Head2, Table S1B). MIS12C^ΔHead2^ retained CENP-C binding (see below) and readily crystallized with it in the absence of proteases. We determined the structure at 3.25 Å resolution as described in the STAR Methods (see also Table 1). The Head1 and Stalk of MIS12C^ΔHead2^ retain the overall organization and relative orientation observed in the crystal of complete MIS12C, except for a small rigid body movement of Head1 relatively to the stalk (Figure 1C). In addition, we report the crystal structure of the isolated Head2 domain determined at 2.0 Å (Figure S2F; Table 1).

#### Interaction of MIS12C with CENP-C

**Figure 2:**
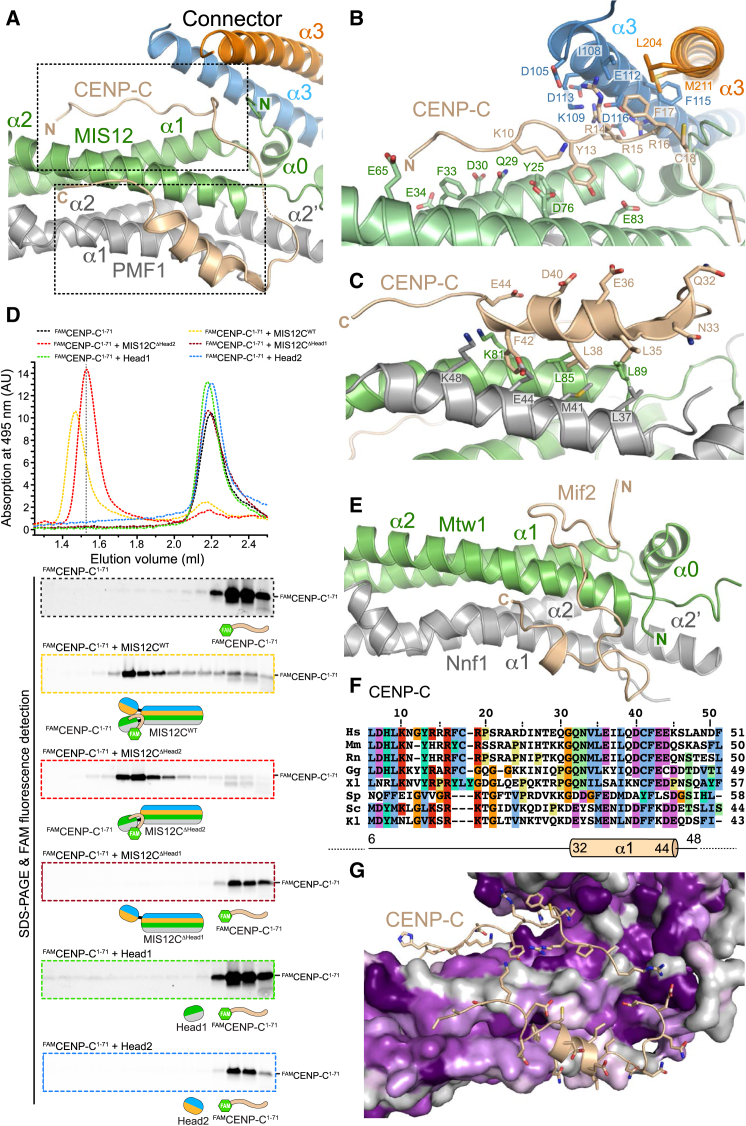
Dissection of MIS12:CENP-C (A) Cartoon model of the interaction of CENP-C^1–71^ with Head1 and the Connector. Two boxed areas are enlarged in (B) and (C). (B) Focus on the N-terminal segment of CENP-C (residues 6–22), with a subset of side chains of residues at the interface. (C) Focus on the amphipathic helix of the CENP-C chain (residues 32–44). (D) SEC analysis of the indicated MIS12C constructs and ^FAM^CENP-C^1–71^. Protein absorption at 280 nm (Figure S3) and FAM absorption at 495 nm (shown) were measured. SEC fractions were analyzed by SDS-PAGE and visualized by Coomassie staining (Figure S3) or FAM fluorescence (shown). A shift to the left in the elution profiles of ^FAM^CENP-C^1–71^ indicates binding to MIS12C. A dotted vertical line indicates elution volume of the MIS12C^ΔHead1^ mutant. (E) Cartoon model of *Kluyveromyces lactis* MIND Head1 bound to Mif2 (Dimitrova et al., 2016). Mtw1 and Nnf1 are MIS12 and PMF1 orthologs, respectively. See Figure S4 for additional structural details. (F) Sequence alignment of the N-terminal region of CENP-C orthologs. Hs, *Homo sapiens*; Mm, *Mus musculus*; Rn, Rattus norvegicus; Gg, *Gallus gallus*; Xl, *Xenopus laevis*; Sp, *Schizosaccharomyces pombe*; Sc, *Saccharomyces cerevisiae*; Kl, *Kluyveromyces lactis*. The alignment was obtained with MAFFT (Katoh and Standley, 2013). (G) Sequence conservation in the CENP-C binding region mapped onto the MIS12C structure. Conservation scores were calculated based on sequences from *Homo sapiens*, *Bos taurus*, *Ovis aries*, *Ornithorhynchus anatinus*, *Gallus gallus*, *Pseudopodoces humilis*, *Python bivittatus*, *Gekko japonicus*, *Xenopus laevis*, *Danio rerio*, *Drosophila busckii*, *Drosophila melanogaster*, *Saccharomyces cerevisiae*, *Kluyveromyces lactis*, and *Schizosaccharomyces pombe*. Sequence conservation is color coded from purple (indicates conserved amino acid positions) to gray (indicates variable amino acid positions).

**Figure S3:**
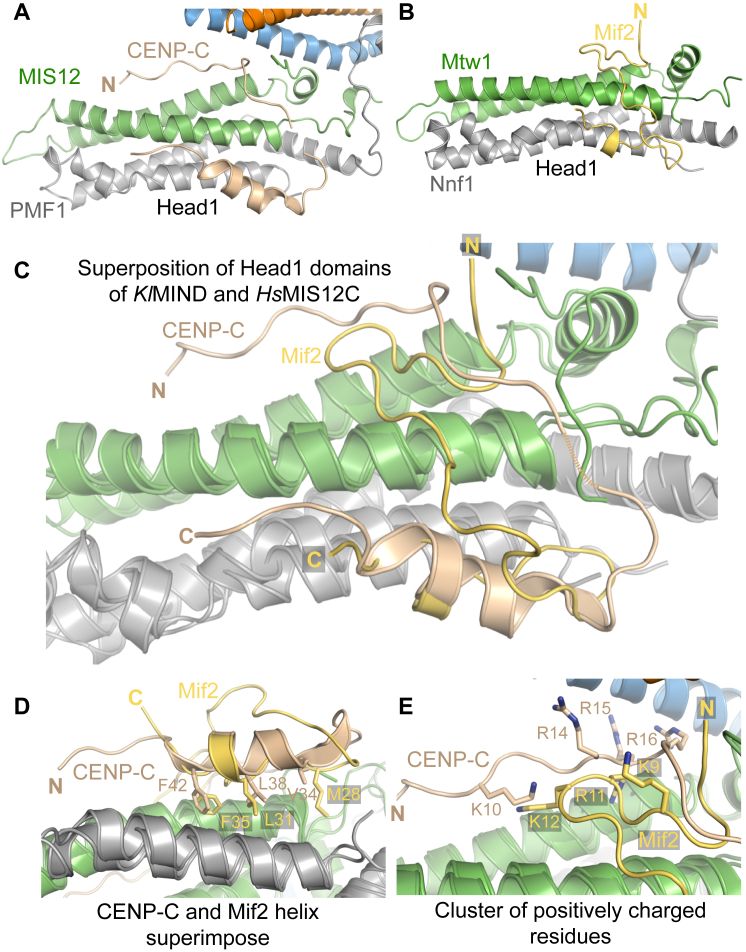
Further Comparison of MIS12C:CENP-C and MIND:Mif2, Related to Figure 2 (A and B) Cartoon diagrams of MIS12C:CENP-C and MIND:Mif2 (left and right, respectively) around Head1 and the CENP-C/Mif2 binding sites. Mif2 is shown here in yellow rather than wheat to facilitate interpretation of subsequent panels. The main chain of CENP-C resembles a “horseshoe.” Its first visible segment (residues 6-22) is extended and binds in a shallow groove between the α1 and α2 helices of MIS12 in Head1. Lys10^CENP-C^, Tyr13^CENP-C^, Arg14^CENP-C^, Arg16^CENP-C^, and Phe17^CENP-C^ interact with residues in Head1 and with the N-terminal region of the α3 helices of DSN1:NSL1 (in the helical connector, see Figure 2B). Asp105^Nsl1^, Glu112^Nsl1^, and Asp113^Nsl1^ in the NSL1 α3 helix are very well conserved in evolution and interact with the side chains of Arg14^CENP-C^, Arg15^CENP-C^, and Arg16^CENP-C^. Lys10^CENP-C^ and Tyr13^CENP-C^ are necessary for tight binding of CENP-C to MIS12C (Screpanti et al., 2011). The CENP-C main chain takes a turn around residues Phe17^CENP-C^ and Cys18^CENP-C^, moving away from the stalk in an extended and poorly conserved segment. Electron density for this segment of CENP-C is weak. The CENP-C chain bends again to complete its “U-turn” around residues 28-30, emerging in helical conformation (residues 32-44, Figure 2C). The CENP-C helix packs snugly against the groove between α1 of PMF1 and α2 of MIS12, and is amphipathic, with the side chains of Val 34^CENP-C^, Leu35^CENP-C^, Ile37^CENP-C^, Leu38^CENP-C^, Cys41^CENP-C^, and Phe42^CENP-C^ pointing inward toward Head1, and those of Glu36^CENP-C^, Asp40^CENP-C^, and Glu44^CENP-C^, pointing outward. (C) Overall superposition of Head1 domains in the two structures demonstrates superposition of the CENP-C and Mif2 helical region, but not of the N-terminal regions. (D) Zoom-in view of the helical region highlighting the similarity of binding mode of CENP-C and Mif2 to Head1 domain. (E) Zoom-in view of the N-terminal regions of CENP-C and Mif2 demonstrates clustering of positively charged residues despite an overall different path of the polypeptide chains on the Head1 surface.

The sequence of the N-terminal region of CENP-C in distant orthologs seems quite divergent, but the function of this region as a MIS12C binding interface is conserved (Hornung et al., 2014, Liu et al., 2016, Przewloka et al., 2011, Richter et al., 2016, Screpanti et al., 2011). The sturdy arrangement of helices in Head1 and in the helical connector of DSN1:NSL1 provides a composite binding site for CENP-C (Figure 2A). The main chain of CENP-C resembles a “horseshoe.” Its first visible segment (residues 6–22) is extended and binds in a shallow groove between the α1 and α2 helices of MIS12 in Head1. Lys10^CENP-C^ and Tyr13^CENP-C^ in this segment are necessary for tight binding of CENP-C to MIS12C (Screpanti et al., 2011). The CENP-C main chain then takes a turn (at Phe17^CENP-C^ and Cys18^CENP-C^) to move away from the stalk through an extended and poorly conserved segment for which there is weak electron density. The CENP-C chain bends again to complete its “U-turn” around residues 28–30, emerging in helical conformation (residues 32–44, Figure 2C; a more detailed description of the interaction is in the legend of Figures S3A and S3B). In both complexes in the asymmetric unit of the MIS12C^ΔHead2^:CENP-C^1-71^ crystals, clear electron density is only visible for residues 6–18 of CENP-C, whereas no density for the helical C-terminal segment is visible, probably due to crystal contacts involving MIS12C. As discussed in the context of Figure 4, the N-terminal region of CENP-C is sufficient for a high-affinity interaction with MIS12C.

#### Validation of the MIS12:CENP-C Interaction

CENP-C binds MIS12C mainly on Head1 but also exploits the interface of Head1 with the helical connector (αC helices of DSN1 and NSL1). We took three steps to validate this binding mode. First, we asked if Head1 was necessary, and possibly sufficient, for CENP-C binding to MIS12C. Second, we asked if the relative position of Head1 and the DSN1:NSL1 helical connector is necessary for high-affinity binding of CENP-C to MIS12C. Finally, we asked to what extent specific residues of MIS12C seen at the interface with CENP-C contribute to the interaction.

**Figure S4:**
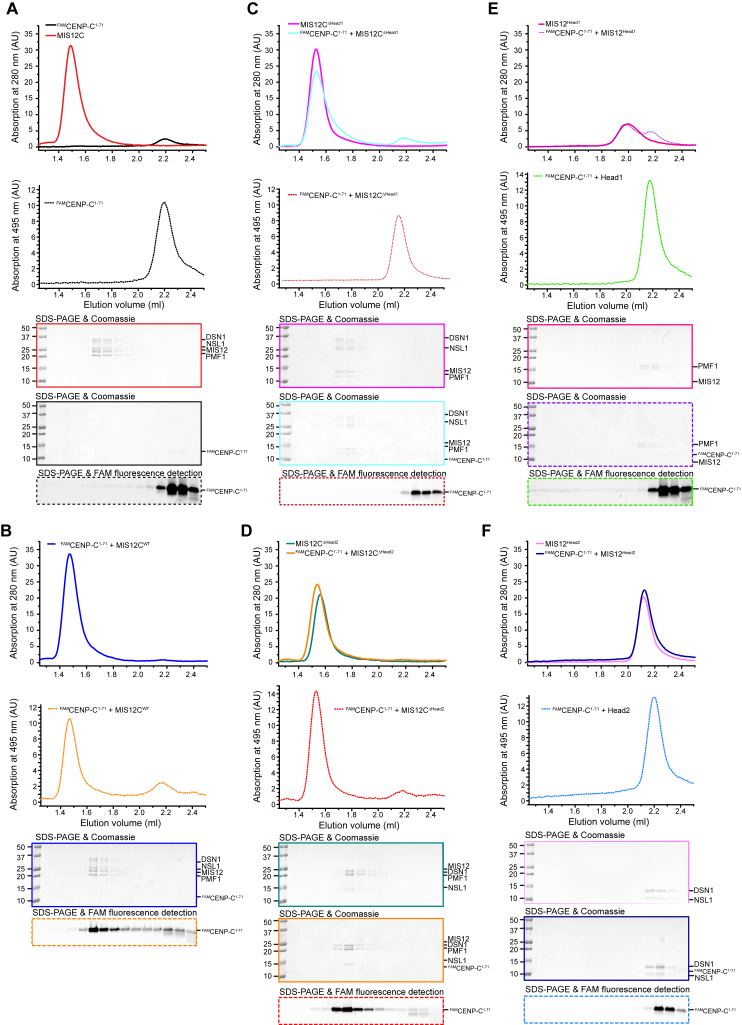
Size-Exclusion Chromatography Assays with SDS-PAGE, Related to Figure 2 (A–F) SEC elution profiles of the indicated species were monitored at 495 nm (to follow FAM absorption) and 280 nm (to follow general protein absorption). SDS-PAGE were analyzed for fluorescence (from ^FAM^CENP-C^1-71^) and also stained with Coomassie Brilliant Blue to visualize all proteins.

To monitor the interaction of CENP-C with MIS12C, we used site-specific Sortase-mediated ligation (Popp and Ploegh, 2011) to create fluorescein amidite (FAM)-labeled CENP-C^1–71^ (^FAM^CENP-C^1–71^). We visualized the interaction of ^FAM^CENP-C^1–71^ with MIS12C through analytical size-exclusion chromatography (SEC) experiments, in which proteins are separated based on their size and shape. We monitored elution of ^FAM^CENP-C^1–71^ from the SEC column by following its absorbance at 495 nm. After separation by SDS-PAGE, we visualized it through fluorescence of its FAM group (Figure 2D). Isolated ^FAM^CENP-C^1–71^ or its complex with MIS12C^Nano^ (herewith also indicated as MIS12C^WT^, to indicate that it did not contain additional mutations) had clearly distinct elution volumes, providing a simple test for assessing the interaction (Figures 2D and S4). A MIS12C construct lacking Head1 (MIS12C^ΔHead1^) did not interact with CENP-C, while MIS12C^ΔHead2^ bound CENP-C like MIS12C^WT^ (even with higher affinity, as explained below). We therefore asked if the isolated Head1 domain bound CENP-C^1–71^, but found this not to be the case at the low micromolar protein concentrations of this assay. Thus, Head1 is necessary, but not sufficient, for high-affinity binding of MIS12C to CENP-C. As expected, we saw no binding of Head2 to CENP-C (Figures 2D and S4).

#### Conservation of MIS12C CENP-C Binding Mode in Eukaryotes

In the accompanying paper, Y.D., S.J., and S.C.H. report the structural and biochemical characterization of the interaction of the fungal (*Kluyveromyces lactis*) MIND complex with the N-terminal region of Mif2 (equivalent to human MIS12C and CENP-C, respectively) (Dimitrova et al., 2016). Unlike the human proteins, a Head1 construct of the MIND complex was sufficient to bind Mif2 and co-crystallized with it. A crystal structure of the Head1:Mif2 complex (Figure 2E) shows that a helical segment of *Kl*Mif2 (residues 30–35), although shorter than that of human CENP-C, superposes well with it. Structural alignment of the helical regions brought to light additional sequence conservation in the N-terminal segment of CENP-C:Mif2, corresponding to residues 6–16 of human CENP-C (Figure 2F). Although the paths of the N-terminal region of CENP-C and Mif2 on Head1 are different (Figures 2A and 2E), in both cases there is clustering of positively charged residues (positions 14 and 16 of human CENP-C) close to the interface of Head1 with the helical connector (Figure S3). Thus, comparison of fungal and mammalian orthologs shows previously unappreciated sequence and structural similarities in the N-terminal region of CENP-C. Evolutionary conservation of residues at the surface of MIS12C, however, is limited (Figure 2G).

#### Role of the *α0* Helix of MIS12 in CENP-C Binding

**Figure 3:**
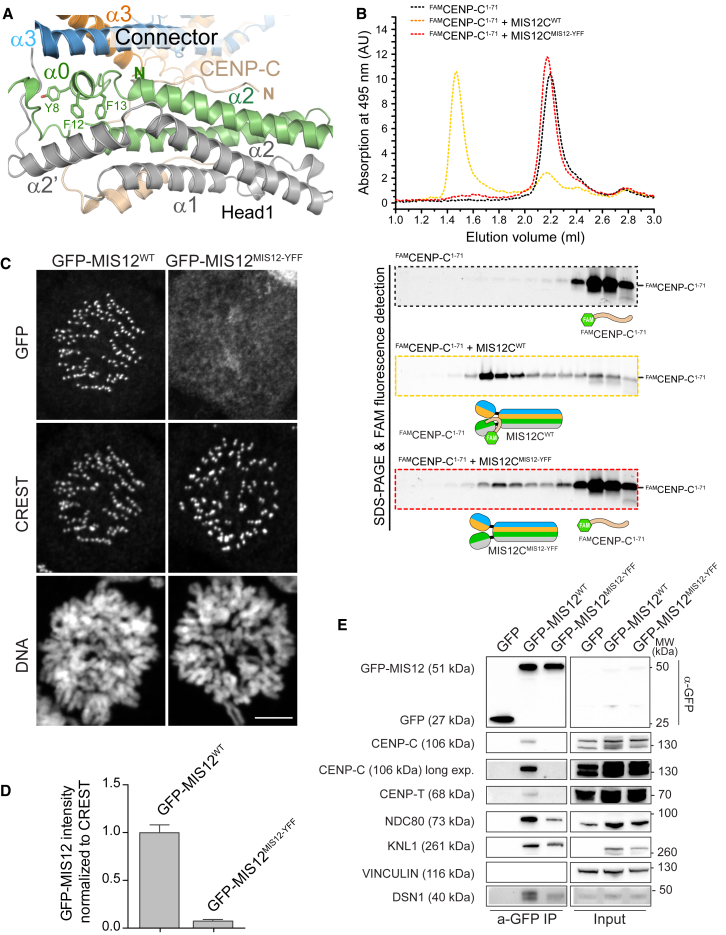
Role of the MIS12 α0 Helix in CENP-C Binding (A) Role of three conserved aromatic residues in the α0 helix of MIS12. (B) The interaction of MIS12C^YFF^ with ^FAM^CENP-C^1–71^ was analyzed by SEC. Control profiles for MIS12C^WT^:^FAM^CENP-C^1–71^ is the same already shown in Figure 2D. The elution profile of MIS12C^WT^ is shown in Figure S4A. Data for absorption at 280 nm and Coomassie staining of SDS-PAGE are shown in Figure S3. (C) Representative images of stable Flp-In T-REx cells expressing the indicated GFP-MIS12 constructs, showing that the YFF mutant does not localize to kinetochores (CREST is an inner kinetochore marker). Scale bar, 10 μm. (D) Quantification of GFP-MIS12 kinetochore levels. The graph shows mean intensity from two independent experiments. Error bars represent SEM. Values for Mis12^WT^ are set to 1. (E) Western blot of immunoprecipitates (IP) from mitotic Flp-In T-REx cell lines expressing the indicated GFP-Mis12 constructs. Vinculin was used as loading control. See also Figure S5.

**Figure S5:**
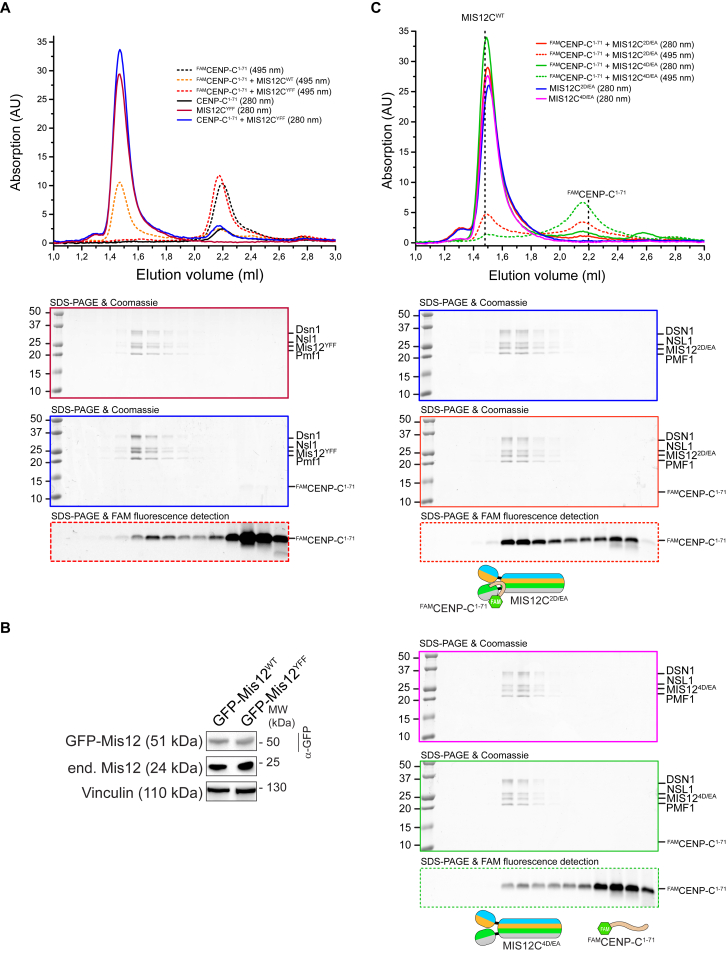
Size-Exclusion Chromatography Assays with SDS-PAGE Loading Controls, Related to Figures 3 and 4 (A and C) SEC analysis of the indicated species with absorbance at 495 nm (to follow FAM absorption) and 280 nm (to follow general protein absorption). SDS-PAGE were analyzed for fluorescence (from ^FAM^CENP-C^1-71^) and also stained with Coomassie Brilliant Blue to visualize all proteins. (B) Western blot of stable Flp-In T-REx cells expressing the indicated MIS12 constructs showing that expression levels of MIS12^WT^ and MIS12C^YFF^ are similar to one another. Vinculin was used as loading control.

Binding of CENP-C to MIS12C occurs at the interface of Head1 with the helical connector, which is stabilized by the α0 helix of MIS12 (Figure 3A). The sequence of the α0 helix of MIS12 is conserved in evolution, especially residues Tyr8^MIS12^, Phe12^MIS12^, and Phe13^MIS12^. The side chains of these residues are buried at the interface of α0^MIS12^ helix with the helical connector (Figure 3A) and are therefore likely to stabilize the orientation of Head1 with respect to the connector. We generated a mutant carrying alanine point mutations of Tyr8^MIS12^, Phe12^MIS12^, and Phe13^MIS12^ (indicated as MIS12C^MIS12-YFF^ mutant). MIS12C^YFF^ was soluble and apparently stable, and its SEC elution profile was essentially undistinguishable from that of MIS12C^WT^ (Figures S4A and S5A). Nonetheless, while ^FAM^CENP-C^1–71^ co-eluted with MIS12C^WT^ from a SEC column, it bound only very poorly to the MIS12C^MIS12-YFF^ mutant (Figures 3B and S5A). These observations corroborate the idea that residues in the α0 helix of MIS12 stabilize the specific packing of Head1 against the helical connector required for high-affinity binding of CENP-C.

To probe the effects of the MIS12C^MIS12-YFF^ mutant on MIS12C kinetochore localization in vivo, we expressed in HeLa cells a chimeric construct of GFP with MIS12C^WT^ or MIS12C^MIS12-YFF^ (Figure S5B). GFP-MIS12^WT^ strongly decorated kinetochores, as shown by co-localization with CREST anti-centromere antibodies, while GFP-MIS12C^MIS12-YFF^, which did not bind to CENP-C in vitro, failed to do so (Figure 3C, quantified in Figure 3D). In immunoprecipitation experiments with an anti-GFP antibody, GFP-MIS12C^WT^ bound endogenous CENP-C, CENP-T, NDC80, and KNL1. Conversely, GFP-MIS12C^MIS12-YFF^ did not appear to interact with CENP-C or CENP-T, while it appeared to interact with other KMN components, but less robustly than GFP-MIS12C^WT^ (Figure 3A). These observations confirm the importance of the α0 helix of MIS12 in stabilizing the interaction of MIS12C with CENP-C and suggest that their association enhances the interactions of MIS12C with other kinetochore proteins, including other KMN sub-complexes.

#### Role of Head1 Negative Charges in CENP-C Binding

**Figure 4:**
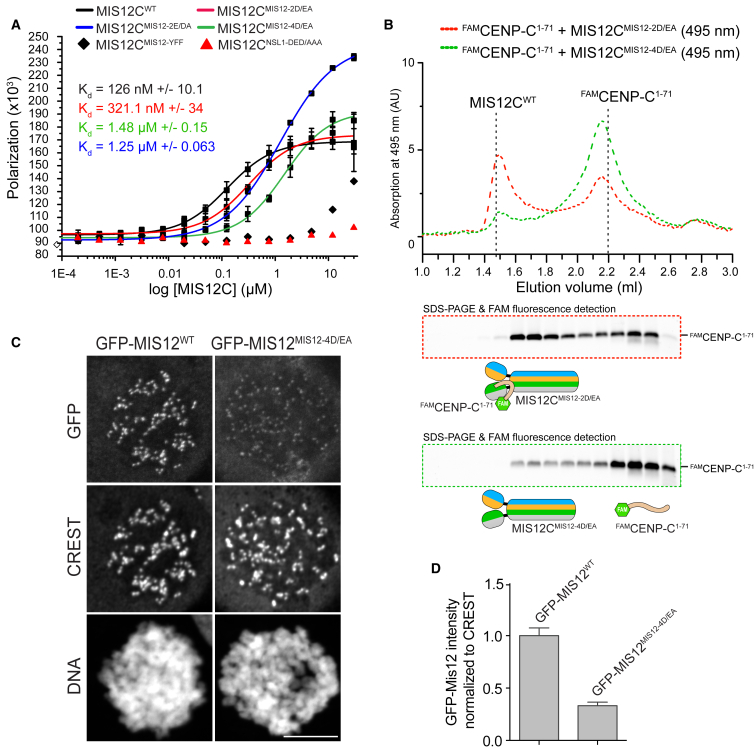
Head1 and the Connector Promote CENP-C Binding (A) Fluorescence polarization experiments with a synthetic ^FAM^CENP-C^1–21^ peptide (at 20 nM concentration). Increasing concentrations of the indicated MIS12C species were added and fluorescence polarization monitored at equilibrium. Data fitting was performed as described in the STAR Methods. Due to the very low binding affinity, binding data for the MIS12C^MIS12-YFF^ and MIS12C^NSL1-EDEAAA^ mutants were not fitted and appear therefore as disconnected points. (B) SEC profiles of the indicated mutant MIS12Cs incubated with ^FAM^CENP-C^1–71^. Dotted vertical bars indicate elution volumes of MIS12C^WT^ and ^FAM^CENP-C^1–71^. (C) Representative images of stable Flp-In T-REx cells expressing the indicated GFP-MIS12 constructs and showing that the GFP-MIS12^MIS12-4D/EA^ mutant is severely impaired in its localization to kinetochores. Scale bar, 10 μm. (D) Quantification of GFP-MIS12 kinetochore levels. The graph shows mean intensity from two independent experiments. Error bars represent SEM. Values for Mis12^WT^ are set to 1. See also Figure S5.

In fluorescence polarization experiments, MIS12C^WT^ bound a 21-residue synthetic fluorescent peptide encompassing the N-terminal region of CENP-C (^FAM^CENP-C^1–21^) with a dissociation constant (K_d_) of 126 nM (Figure 4A). Thus, even if this peptide lacks residues in the CENP-C helical segment (residues 32–44), it binds MIS12C with high affinity, in line with previous data (Screpanti et al., 2011). We used this assay to test the effects of alanine point mutations in conserved residues of Head1 involved in the interaction with the N-terminal region of CENP-C. MIS12C with Asp30^MIS12^ and Glu34^MIS12^ mutated to alanine (MIS12C^MIS12-2D/EA^) bound ^FAM^CENP-C^1–71^ in analytical SEC experiments, but at reduced levels (Figures 4B and S5C). The affinity of MIS12C^MIS12-2D/EA^ for ^FAM^CENP-C^1–21^ was almost 3-fold lower than that of wild-type, but a K_d_ of 321 nM was strong enough for co-elution in SEC experiments. A different two-alanine variant of MIS12C (MIS12C^MIS12-2E/DA^) with Glu65^MIS12^ and Asp76^MIS12^ in the MIS12 subunit mutated to alanine, or a four-alanine variant Asp30^MIS12^, Glu34^MIS12^, Glu65^MIS12^, and Asp76^MIS12^ all mutated to alanine, had more severe effects. MIS12C^MIS12-4D/EA^ showed a severely disrupted association with ^FAM^CENP-C^1–71^ in analytical SEC experiments (Figure 4B). Its K_d_ for ^FAM^CENP-C^1–21^ was 1.48 μM, while that of MIS12C^MIS12-2E/DA^ for ^FAM^CENP-C^1-21^ was 1.25 μM (Figure 4A). In agreement with these observations, kinetochore recruitment of a GFP-MIS12^4E/DA^ construct was severely impaired (Figures 4C and 4D). We also created a triple alanine mutant of residues Asp105^Nsl1^, Glu112^Nsl1^, and Asp113^Nsl1^, three evolutionary conserved residues in the NSL1 α3 helix that interact with the side chains of Arg14^CENP-C^, Arg15^CENP-C^, and Arg16^CENP-C^ (Figure 2B). The resulting mutant, MIS12C^NSL1-DEDAAA^, was unable to bind ^FAM^CENP-C^1–21^ in fluorescence polarization experiments (Figure 4A). Thus, the results of the mutagenesis analysis confirm our inference from the structure that conserved acidic residues of MIS12 and NSL1 participate in binding the N-terminal region of CENP-C.

#### Role of HEAD2 Phosphorylation in CENP-C Binding to MIS12C

**Figure 5:**
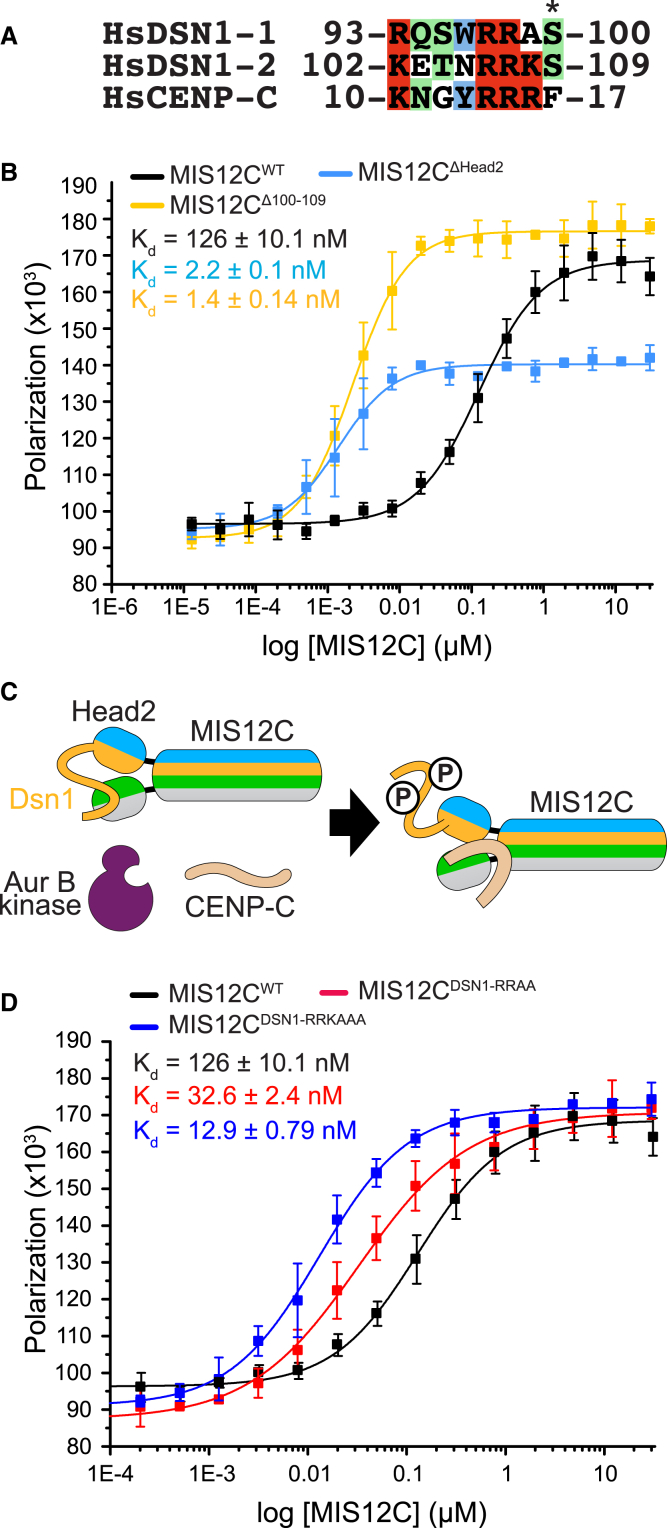
Intramolecular Regulation of CENP-C Binding (A) Sequence motifs in DSN1 that are phosphorylated by Aurora B aligned with a segment in the N-terminal region of CENP-C. (B) Fluorescence polarization experiments were carried out as already shown in Figure 4A with the indicated MIS12C species. (C) Scheme detailing how Aurora B may regulate binding of CENP-C to MIS12C. (D) Fluorescence polarization experiments were carried out as already shown in (B) with the indicated MIS12C mutant complexes. See also Figure S6.

Aurora B phosphorylates DSN1 at Ser100^DSN1^ and Ser109^DSN1^ (Welburn et al., 2010, Yang et al., 2008). Being preceded by positively charged residues at the −3 and −2 positions, Ser100^DSN1^ and Ser109^DSN1^ are ideal phosphorylation substrates of Aurora B kinase (Figure 5A). Phosphorylation of Ser100^DSN1^ and Ser109^DSN1^ stabilizes the interaction of MIS12C with kinetochores, and short deletions encompassing the DSN1 region containing Ser100^DSN1^ and Ser109^DSN1^ rescue the detrimental effects of Aurora B inhibition on kinetochore assembly (Kim and Yu, 2015, Rago et al., 2015). Thus, phosphorylation of Ser100^DSN1^ and Ser109^DSN1^ may only be required to remove an inhibitory effect of unphosphorylated DSN1 on the interaction of the MIS12C with CENP-C, rather than playing a positive role on CENP-C binding after phosphorylation. Indeed, the MIS12C^ΔHead2^ construct, which also lacks Ser100^DSN1^ and Ser109^DSN1^, binds CENP-C in SEC experiments (Figure 2D). Thus, Head2 and the segments that precede it (also deleted in the MIS12C^ΔHead2^ construct, see Table S1B) are not required for CENP-C binding. Indeed, MIS12C^ΔHead2^ bound ^FAM^CENP-C^1-21^ in fluorescence polarization experiments with a K_d_ of 2.2 nM, i.e., almost 60-fold more tightly than MIS12C^WT^ (Figure 5B). We observed an equivalent increase in affinity with a shorter deletion mutant of MIS12C, which lacked only the 10-residue segment encompassing Ser100^DSN1^ and Ser109^DSN1^ (MIS12C^DSN1Δ100–109^, Figure 5B). These results argue that segment 100–109 of DSN1 likely binds directly to the CENP-C binding site of MIS12C through an inter-subunit interaction, as depicted schematically in Figure 5C. Aurora B phosphorylation of DSN1 relieves this inhibitory effect, possibly to focus the interaction of MIS12C with CENP-C to kinetochores, where Aurora B activity concentrates during mitosis (Akiyoshi et al., 2013, Caldas et al., 2013, Liu et al., 2009, Welburn et al., 2010).

**Figure S6:**
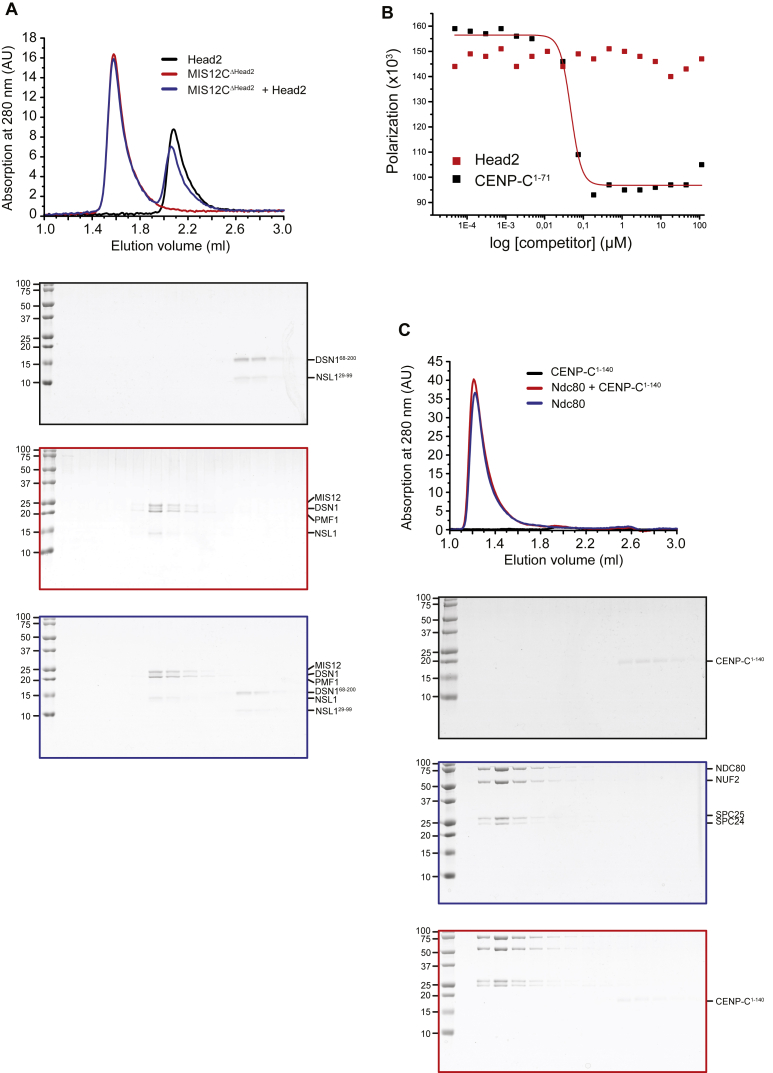
Additional Binding Assays, Related to Figures 5 and 6 (A and C) SEC analysis of the indicated species with absorbance at 280 nm. SDS-PAGE were stained with Coomassie Brilliant Blue to visualize all proteins. (B) Fluorescence polarization experiments were carried out with a synthetic ^FAM^CENP-C^1-21^ peptide and saturating concentrations of the indicated MIS12C species and polarization monitored at equilibrium (like in Figure 4A). Unlabeled CENP-C^1-71^ was added as positive control for competition at the indicated competitor concentration. Head2 had no effects as competitor even at high concentrations, suggesting that its intra-molecular interaction with Head1 is of very modest affinity.

The motifs of HsDSN1 encompassing Ser100^DSN1^ and Ser109^DSN1^ are related and can be also tentatively aligned with residues 10–17 of HsCENP-C (Figure 5A), suggesting that DSN1 interferes with the interaction of the N-terminal region of CENP-C with MIS12C. This hypothesis agrees with the results of our assays in Figure 5B demonstrating the effects of DSN1 on CENP-C binding, as these were carried out with ^FAM^CENP-C^1–21^, i.e., with a peptide encompassing residues 10–17 of CENP-C. To gain stronger evidence for this hypothesis, we created alanine mutants of residues Arg106^DSN1^, Arg107^DSN1^, and Lys108^DSN1^, and measured binding affinity for ^FAM^CENP-C^1–21^ by fluorescence polarization. In agreement with the hypothesis, MIS12C^DSN1-RRAA^ and MIS12C^DSN1-RRKAAA^ bound ^FAM^CENP-C^1–21^ with progressively higher binding affinity, implicating positively charged residues in the DNS1-2 motif in the mechanism of intra-molecular regulation (Figure 5D). A construct comprising both Ser100^DSN1^ and Ser109^DSN1^ and Head2 did not bind directly to MIS12C^ΔHead2^ (Figure S6A), nor it displaced, even at concentrations in excess of 100 μM, ^FAM^CENP-C^1–21^ from MIS12C^ΔHead2^, whereas a competitor CENP-C^1–71^ peptide competed with ^FAM^CENP-C^1–21^ effectively (Figure S6B). We therefore surmise that the high effective concentration of the DSN1 segment responsible for the regulation of the interaction of MIS12C with CENP-C compensates for a low interaction affinity.

#### Interaction of MIS12C with NDC80C and KNL1C

**Figure 6:**
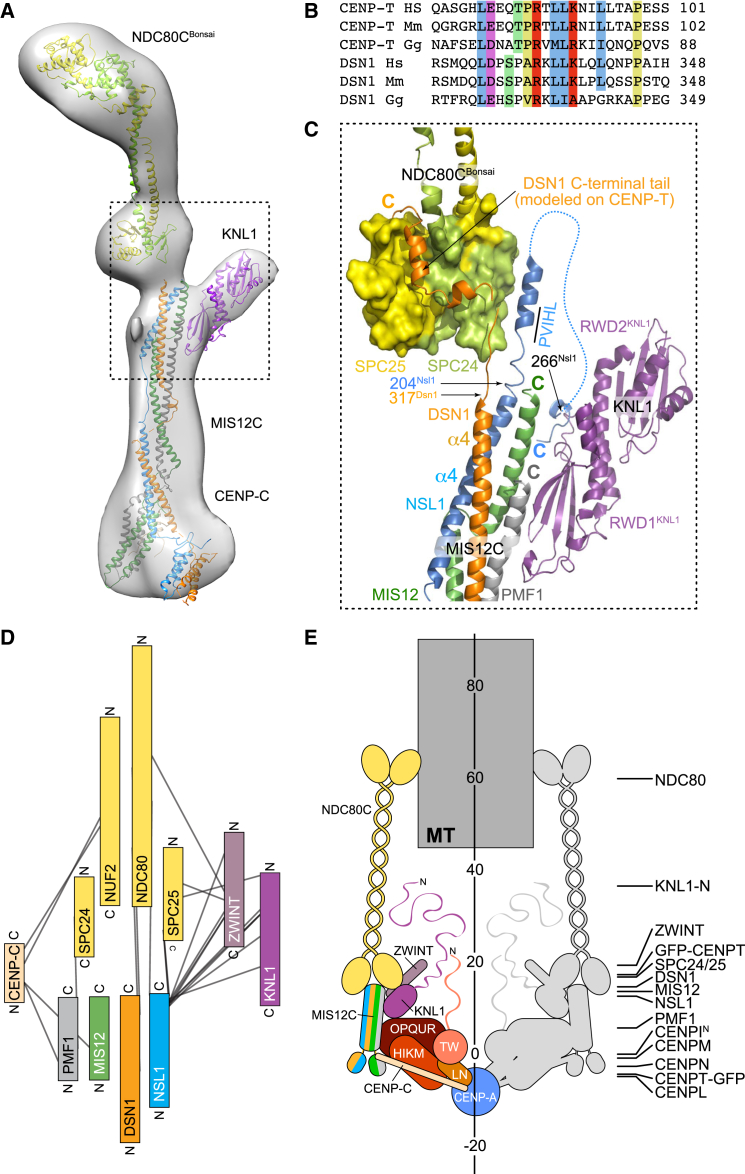
KMN Assembly and Wider Kinetochore Organization (A) Chimera (Pettersen et al., 2004) was used to fit model of MIS12C in a 3D negative stain EM map (EMD-2549) of a nine-subunit complex containing MIS12C, NDC80C^Bonsai^, and the C-terminal RWD domains of KNL1 (Petrovic et al., 2014). (B) Sequence alignment of the SPC24:SPC24-binding region in the N-terminal region of CENP-T and of the C-terminal region of DSN1. (C) The C-terminal region of DSN1 downstream of the terminal part of the stalk domain was modeled on the structure of the SPC24:SPC25:CENP-T complex (PDB: 3VZA). The PVIHL motif is necessary for SPC24:SPC25 binding and is predicted to start a helical segment of NSL1. The chain then inverts direction to reach KNL1. The structure of the KNL1 RWD domains bound to the NSL1 C-terminal peptide (PDB: 4NF9) identifies the peptide at the junction between RWD domains (Petrovic et al., 2014). (D) The diagram illustrates the intermolecular cross-links connecting subunits in different KMN complexes (listed in part A of Table S2). (E) Schematic view of kinetochores drawn with “complexes” with realistic relative scales. The gray moiety may be generated by pseudo 2-fold symmetry of the CENP-A nucleosome (Weir et al., 2016). A ruler (in nanometers) was positioned along the inter-kinetochore axis. On the right, we indicate the coordinate along the inter-kinetochore axis of the centroid of a fluorescence signal associated with the indicated proteins (e.g., by fusion to GFP or through antibodies). The zero coordinate was arbitrarily assigned to CENP-I^N^ (the N terminus of CENP-I) (Suzuki et al., 2014, Wan et al., 2009). See also Figure S6.

The crystal structure of the MIS12C fits snugly into a 3D negative stain-EM reconstruction (Figure 6A) (Petrovic et al., 2014) of an artificial KMN assembly construct consisting of MIS12C, the tandem RWD (RING finger, WD repeat, DEAD-like helicases) domains in the C-terminal region of KNL1 (Petrovic et al., 2014), and NDC80C^Bonsai^, an engineered chimeric construct of the NDC80C (NDC80C^Bonsai^) in which most of the coiled-coil regions of NDC80C had been removed to facilitate crystallization (Ciferri et al., 2008).

Two sequence motifs in human DSN1 and NSL1 (encompassing residues 323–348 and 209–213, respectively) have been previously implicated in the interaction of the MIS12C with the SPC24 and SPC25 subunits of NDC80C (that also consist of RWD domains) (Malvezzi et al., 2013, Petrovic et al., 2010). The NSL1 and DSN1 motifs immediately follow the last visible residues of the DSN1 and NSL1 subunits in the crystal structure of MIS12C (317^DSN1^ and 204^NSL1^, respectively). We can infer the mode of NDC80C binding by the DSN1 motif on the basis of its sequence similarity to the NDC80C-binding motif of CENP-T (known as Cnn1 in *S. cerevisiae*) (Figure 6B). A crystal structure of Cnn1 in complex with the SPC24:SPC25 dimer has been previously determined (Malvezzi et al., 2013, Nishino et al., 2013), and we can therefore model the DSN1:NDC80 interaction on the experimental structure of the CENP-T^Cnn1^:SPC24:SPC25 complex (PDB: 3VZA), as shown in Figure 6C. The accompanying paper also includes a crystal structure of the yeast Spc24:Spc25 heterodimer with the C-terminal moiety of Dsn1 (Dimitrova et al., 2016).

We can also infer the mode of NDC80C binding by the NSL1 motif (Petrovic et al., 2010) on the basis of secondary structure predictions (with the PSIPRED server; Buchan et al., 2013) suggesting that the NSL1 segment 209-PVIHLQRIHQEVFS-222 adopts helical conformation. We speculate that this segment of NSL1, which is necessary but not sufficient for binding of human MIS12C and NDC80C (Petrovic et al., 2010), extends the stalk, making contacts with the RWD domains of SPC24:SPC25 (Figure 6C). The NSL1 chain then likely reverses its direction to reach the KNL1 RWD domains, to which it binds through a motif comprised between NSL1 residues 258 and 281 (Petrovic et al., 2010, Petrovic et al., 2014). Our previously reported crystal structure of this interaction (PDB: 4NF9) showed that the NSL1 C-terminal tail binds at the interface of the RWD domains (Petrovic et al., 2014). Fitting of the EM map, however, shows that KNL1 establishes a much more extended interface with the C-terminal four-helix bundle in the stalk of MIS12C (Figure 6C). While at the resolution of the EM map, no detailed molecular description of this interface is possible, our model explains why the binding affinity of KNL1 for the NSL1 C-terminal peptide is significantly lower than the binding affinity of KNL1 for the entire MIS12C (Petrovic et al., 2010, Petrovic et al., 2014). Furthermore, extensive cross-linking between ZWINT and the C-terminal tail of NSL1 (Figure 6D; Tables S2 and S3; for these experiments we used the full length sequences of MIS12C subunits) suggests that ZWINT, the only remaining KMN subunit to remain structurally uncharacterized, also positions itself near the KNL1 binding site of NSL1, in agreement with its ability to interact directly with the KNL1 C-terminal region (Petrovic et al., 2014). The cross-linking analysis also identified possible contacts between the NDC80C and CENP-C^1–140^, but this species, at 5 μM, did not show a direct interaction by SEC (Figure S6C).

## Discussion

Together with previous high-resolution structural analyses of NDC80C and KNL1 (Ciferri et al., 2008, Malvezzi et al., 2013, Nishino et al., 2013, Petrovic et al., 2014, Primorac et al., 2013, Wei et al., 2006, Wei et al., 2007), the crystal structures of Mis12C reported here, and its yeast ortholog, MIND, described in the accompanying paper (Dimitrova et al., 2016), represent an important step toward the generation of a comprehensive model of the KMN assembly. These high-resolution analyses complement sub-nanometer resolution 3D cryo-EM reconstructions of NDC80C^Bonsai^ on microtubules, which revealed how the two tightly arranged calponin homology (CH) domains in the NDC80 and NUF2 subunits of NDC80C and a disordered N-terminal segment of NDC80 cooperate for high-affinity microtubule binding (Alushin et al., 2010).

Besides DSN1 (as discussed in Results), all CCAN subunits, except CENP-C, seem to have been lost from the genomes of *Drosophila melanogaster* and of a few other organisms (Barth et al., 2014, Drinnenberg et al., 2014, Meraldi et al., 2006, Przewloka et al., 2007, Westermann and Schleiffer, 2013). As in humans and yeast, the interaction of MIS12C with CENP-C in *Drosophila* engages the N-terminal region of CENP-C (Hornung et al., 2014, Liu et al., 2016, Przewloka et al., 2011, Richter et al., 2016, Screpanti et al., 2011). This linkage may be the only connection between the inner and outer kinetochore in organisms devoid of additional CCAN subunits. In organisms that contain CCAN, on the other hand, a pathway of outer kinetochore recruitment centered on the CCAN subunit CENP-T (Cnn1 in *S. cerevisiae*) acts in parallel to the CENP-C pathway to promote KMN recruitment (Gascoigne et al., 2011, Hori et al., 2008, Kim and Yu, 2015, Malvezzi et al., 2013, Nishino et al., 2013, Rago et al., 2015, Schleiffer et al., 2012, Suzuki et al., 2015). Understanding how the CENP-C and CENP-T pathways co-exist and possibly cooperate in kinetochore assembly is a challenge for future studies.

In Figure 6E, the KMN and CCAN components are drawn schematically but at their approximate scale. CENP-A is assumed to be in axis with the microtubule. Its closest CCAN components are CENP-C and CENP-LN, previously shown to bind CENP-A directly (Carroll et al., 2009, Carroll et al., 2010, Kato et al., 2013). Most of CENP-C is disordered and flexible, with the exception of its C-terminal Cupin-like dimerization domain (Cohen et al., 2008) (not shown in Figure 6). Besides binding CENP-A (residues 516–537 of CENP-C) (Kato et al., 2013), CENP-C binds the CCAN complexes CENP-LN and CENP-HIKM (residues 189–400 of CENP-C) (Klare et al., 2015, Nagpal et al., 2015, Weir et al., 2016) and MIS12C through its N-terminal region (Hornung et al., 2014, Liu et al., 2016, Przewloka et al., 2011, Richter et al., 2016, Screpanti et al., 2011, Weir et al., 2016). The ordered succession of binding sites on CENP-C appears to recapitulate the outer to inner kinetochore axis, suggesting that it is a scaffold ordering kinetochore assembly (Klare et al., 2015). The additional CCAN subunits that interact with CENP-C or, more generally, whose localization to kinetochores depends on CENP-C, including CENP-TW and CENP-OPQUR, have been also implicated in MIS12C localization (Hornung et al., 2014, Kim and Yu, 2015, Rago et al., 2015). At least in fungi, a direct interaction with the MIND complex of the homologs of the CENP-OPQUR subunits has been identified (Dimitrova et al., 2016, Hornung et al., 2014), but we could not detect this interaction with the human proteins (unpublished data).

In summary, CCAN, using CENP-C as a spacer, may generate sturdy linkages radiating from CENP-A to position multiple KMN assemblies to surround a microtubule. The arrangement of CCAN and KMN complexes in Figure 6 fits well with the positioning of individual subunits by a pseudo-super resolution analysis (Joglekar et al., 2009, Suzuki et al., 2014, Wan et al., 2009). These studies and the model derived here are consistent with the observed kinetochore thickness (∼80 nm), most of which can be spanned by the KMN assembly.

## STAR★Methods

### Key Resources Table

**Table:** 

REAGENT or RESOURCE	SOURCE	IDENTIFIER
**Antibodies**		

Rabbit polyclonal anti-GFP	Generated in-house	N/A
Mouse monoclonal anti-MIS12	Generated in-house	#QA21-74-4-3
Rabbit polyclonal anti-CENP-C	Trazzi et al., 2009	#SI410
Mouse monoclonal anti-Vinculin	Sigma-Aldrich	Cat#V9131; RRID: AB_477629
Rabbit polyclonal anti-KNL1-N	Generated in-house	#SI0787
Mouse monoclonal anti-HEC1	GeneTex	Cat#GTX70268; RRID: AB_371632
Rabbit polyclonal anti-CENP-T/W	Generated in-house	#SI0882
Mouse monoclonal anti-DSN1	Generated in-house	#RC11-3(40)
Sheep anti-mouse HRP	Amersham	Cat#NXA931-1ML; RRID: AB_772209
Donkey anti-rabbit HRP	Amersham	Cat#NXA934-1ML
Human anti-centromere (CREST)	Antibodies Inc.	Cat#15-234-0001
Goat anti-human Alex Fluor 647	Invitrogen	Cat#A-21445; RRID: AB_2535862

**Chemicals, Peptides, and Recombinant Proteins**		

GST-Prescission (3C protease)	Musacchio Lab	N/A
Endoproteinase Asp-N	Sigma Aldrich	Cat#000000011420488001
Lysyl Endopeptidase	Wako	Cat#125-05061
Ndc80C	Petrovic et al., 2014	N/A
KNL1C	Petrovic et al., 2014	N/A
Zwint1	Musacchio Lab	N/A
Protease-inhibitor mix HP Plus	Serva	Cat#39107
PhosSTOP phosphatase inhibitors	Roche	Cat#04906845001
DNaseI	Roche	Cat#13146700
FAM-CENP-C 1-21	GenScript	N/A
FAM-GGGK	GenScript	N/A
Sortase A (*S.pyogenes*)	Hidde Ploegh Lab	Addgene:Cat#51139
Sortase A delta 59 (*S.aureus*)	Hidde Ploegh Lab	Addgene:Cat#51138
Zeocin	Invitrogen	Cat#R25001
Doxycycline	Sigma	Cat#D9891;
		CAS: 24390-14-5
Nocodazole	Sigma	Cat#M1404;
		CAS: 31430-18-9
L-glutamine	PAN Biotech	P04-80100
DAPI	Serva	Cat#18860.01
Poly-L-Lysine	Sigma Aldrich	Cat#P4832;
		CAS: 25988-63-0
Bissulfosuccinimidylsuberate (BS3)	Creative Molecules	Cat#001SS
Urea	Sigma-Aldrich	Cat#U6504;
		CAS: 57-13-6
4-(4,6-Dimethoxy-1,3,5-triazin-2-yl)-4-methylmorpholinium chloride (DMTMM)	Sigma Aldrich	Cat#74104;
		CAS: 3945-69-5
Acetonitrile	Fluka	Cat#34967;
		CAS: 75-05-8
Trypsin	Promega	Cat#V511
Ammonium Bicarbonate (Ambic)	Fluka	Cat#C990X98;
		CAS: 1066-33-7
Trifluoroacetic acid (TFA)	Sigma Aldrich	Lot#RB228879;
		CAS: 76-05-1
Tris(2-carboxyethyl)phosphine hydrochloride (TCEP-HCl)	Pierce	Cat#20490; CAS: 51805-45-9
Iodoacetamide	Sigma Aldrich	Cat#I6125;
		CAS: 144-48-9

**Critical Commercial Assays**		

JBS Floppy-Choppy	Jena Bioscience	Cat#CO-110
JBS Tantalum Cluster Derivitization Kit	Jena Bioscience	Cat#PK-103
JSCG Core Suites	QIAGEN	Cat#130724;130725;130726;30727

**Deposited Data**		

MIS12/CENP-C full-length structure	This study	PDB:5LSK
MIS12^Δhead2^ structure	This study	PDB:5LSJ
Head2 structure	This study	PDB:5LSI

**Experimental Models: Cell Lines**		

*Trichoplusia ni*:BTI-Tnao38	Garry W Blissard Lab	N/A
*S.frugiperda*:Sf9 cells in Sf900™ III SFM	ThermoFisher	Cat#:12659017
Human: Flp-In T-Rex HeLa	S.S. Taylor, University of Manchester	N/A
Human: Flp-In T-Rex HeLa-MIS12	This paper	N/A
Human: Flp-In T-Rex HeLa-MIS12 YFF	This paper	N/A
Human: Flp-In T-Rex HeLa-MIS12 4D/EA	This paper	N/A

**Experimental Models: Organisms/Strains**		

E.coli:BL21-CodonPlus(DE3)-RIL strain	Agilent Technologies	#230240
E.coli:One Shot OmniMAX 2 T1R Chemically Competent Cells	ThermoFisher	#C854003
E.coli:BL21-CodonPlus(DE3)-RIPL strain	Agilent Technologies	#230280

**Recombinant DNA**		

pGEX-2rbs	Musacchio Lab	
MultiBac	Geneva Biotech	N/A
pST39	Tan et al., 2005	N/A
pST39-MIS12C	This study	N/A
pST39-MIS12C ^YFF^	This study	N/A
pST39-MIS12C ^MIS12-2D/EA^	This study	N/A
pST39-MIS12C ^MIS12-2E/DA^	This study	N/A
pST39-MIS12C ^MIS12-4D/EA^	This study	N/A
pST39-MIS12C ^NSL1-DED/AAA^	This study	N/A
pST39-MIS12C ^Δ100-109^	This study	N/A
pST39-MIS12C ^DSN1-RRAA^	This study	N/A
pST39-MIS12C ^DSN1-RRKAAA^	This study	N/A
pST39-MIS12C ^Δhead1^	This study	N/A
pST39-MIS12C ^Δhead2^	This study	N/A
pGEX-2rbs-Head1	This study	N/A
pGEX-2rbs-Head2	This study	N/A
pGEX-CENP-C ^1-71^	Screpanti et al., 2011	N/A
pCDNA 5/FRT/TO plasmid	Invitrogen	Cat#V6520-20
pcDNA 5/FRT/TO EGFP	Krenn et al., 2012	N/A
pcDNA 5/FRT/TO EGFP-MIS12	This study	N/A
pcDNA 5/FRT/TO EGFP-MIS12 ^YFF^	This study	N/A
pcDNA 5/FRT/TO EGFP-MIS12^4D/EA^	This study	N/A
pFL-MIS12:PMF1	This study	N/A
pUCDM-NSL1:DSN1	This study	N/A

**Software and Algorithms**		

Origin	OriginLab	www.originlab.com
ImageJ 1.46 r	NIH	https://imagej.nih.gov/ij/
Imaris 7.3.4 32-bit	Bitplane	http://www.bitplane.com/imaris
GraphPad Prism 6.0	GraphPad software	http://www.graphpad.com
Coot	Emsley et al., 2010	http://www2.mrc-lmb.cam.ac.uk/personal/pemsley/coot/
UCSF Chimera	Pettersen et al., 2004	https://www.cgl.ucsf.edu/chimera
Phenix	Adams et al., 2010	https://www.phenix-online.org
XDS	Kabsch, 2010	http://xds.mpimf-heidelberg.mpg.de/
PyMol	The PyMOL Molecular Graphics System, Version 1.2r3pre, Schrödinger, LLC	https://www.pymol.org/
UCLA Diffraction Anisotropy Server		http://services.mbi.ucla.edu/anisoscale
CCP4 package	Winn et al., 2011	www.ccp4.ac.uk
*xQuest/XProphet*	Walzthoeni et al., 2012	http://proteomics.ethz.ch/cgi-bin/xquest2_cgi/index.cgi
SHARP	Bricogne et al., 2003	https://www.globalphasing.com

**Other**		

Sep-Pak C18 Vac Cartridge, 50 mg Sorbent	Waters	Cat#WAT054955
Zeba Spin Desalting Columns, 7K MWCO, 0.5 mL	Thermo Scientific	Cat#89883
Amicon concentrators (3K/10K/30K)	Millipore	Cat#UFC900324;UFC901024;UFC903024
Nitrocellulose membrane	GE Healthcare	Cat#10600001
Mowiol mounting media	Calbiochem	Cat#475904
4-12% NuPAGE Bis-Tris gels	Life Technologies	Cat#NP0321BOX
GST-Trap FF (5 ml)	GE Healthcare	Cat#17-5130-01
His Trap FF (5ml)	GE Healthcare	Cat#17-5255-01
Superdex 75 (10/300) GL	GE Healthcare	Cat#17-5174-01
Superdex 75 (16/600) pg	GE Healthcare	Cat#28-9893-33
Superdex 200 (10/300) GL	GE Healthcare	Cat#17-5175-01
Superdex 200 Increase 5/150 GL	GE Healthcare	Cat#28-9909-45
Superdex Peptide PC 3.2/300	GE Healthcare	Cat#29-0362-31
Resource Q (6 ml)	GE Healthcare	Cat#17-1179-01
Resource S (6 ml)	GE Healthcare	Cat#17-1180-01
Glutathione Affinity Resin	Expedeon	Cat#AGS0010
Protein G-agarose beads	Amintra	Cat#APG0005
Protein A-agarose beads	Roth	Cat#1278.1
GFP-Trap_A	ChromoTek	Cat#gta-20
Corning 384 Well Low Volume Black Round Bottom Polystyrene NBS Microplate	Corning	Cat#4514
ECL Prime western blotting system	GE Healthcare	Cat#RPN 2232

### Contact for Reagent and Resource Sharing

Further information and requests for reagents may be directed to, and will be fulfilled by, Andrea Musacchio (andrea.musacchio@mpi-dortmund.mpg.de).

### Experimental Model and Subject Details

cDNAs used for expression of recombinant proteins were either of human origin, or generated synthetically based on human sequences.

Flp-in T-Rex HeLa cell lines were maintained at 37°C and 5% CO_2_ in DMEM supplemented with 10% tetracycline-free FBS, 2 mM L-glutamine. Doxycycline-inducible stable cell lines were generated using the pcDNA 5/FRT/TO-based plasmids (Tighe et al., 2004). Flp-In T-REx HeLa cells used to generate stable doxycycline-inducible cell lines were a gift from S.S. Taylor (University of Manchester, Manchester, England, UK). Flp-In T-REx host cell lines were maintained in DMEM with 10% tetracycline-free FBS supplemented with 50 μg/ml Zeocin. Flp-In T-REx HeLa expression cell lines were generated as previously described (Petrovic et al., 2014). Transgene expression was induced by the addition of 100 ng/ml Doxycycline hydrate for 24 hr.

*E. coli* BL21(DE3)-Codon-plus-RIL (or RIPL) cells were grown in Terrific Broth (TB) at 37°C.

### Method Details

#### Plasmids

The N-terminal EGFP-MIS12 full-length constructs used for in vivo experiments were generated by subcloning in pcDNA5/FRT/TO/EGFP-IRES vector, a modified derivative of the pCDNA 5/FRT/TO plasmid. The pcDNA 5/FRT/TO/EGFP vector was obtained by cloning the sequence encoding EGFP from pEGFP-C1 into the pcDNA 5/FRT/TO-IRES vector. Sequences encoding deletion versions of MIS12 used for in vitro studies were generated using a modified procedure of the standard Quick-Change site-directed mutagenesis kit protocol in pST39 background (Sawano and Miyawaki, 2000). The constructs encoding Head1 and Head2 versions were generated by Gibson assembly method in pGEX-2rbs, a modified derivative of pGEX-6P-1 expression vector generated in-house. Site-directed mutants were introduces using a modified procedure of the standard Quick-Change site-directed mutagenesis kit protocol (Sawano and Miyawaki, 2000). All plasmids were verified by DNA sequencing.

#### Immunoprecipitation and Immunoblotting

For immunoprecipitation, mitotic cells were harvested by shake-off and lysed in buffer (75 mM HEPES pH 7.5, 150 mM KCl, 1.5 mM MgCl_2_ 1 mM EGTA, 10% glycerol, and 0.075% NP-40) supplemented with and protease inhibitor cocktail and PhosSTOP phosphatase inhibitors. For immunoprecipitation experiments, extracts were pre-cleared with a mixture of protein A–agarose and protein G–agarose for 1 hr at 4°C and subsequently incubated with GFP-Traps (3 μl/mg of extract) for 3 hr at 4°C. Immunoprecipitates were washed in lysis buffer and resuspended in sample buffer, boiled at 95°C, resolved on SDS-PAGE with NuPAGE Bis-Tris 4%–12% gradient gels and transferred onto nitrocellulose membranes. Antibody concentrations were as follows anti-GFP, 1:1000-3000; anti-Mis12, 1:1000; anti-CENP-C, 1:500; anti-Vinculin, 1:20000; anti-Knl1-N, 1:1000; anti-Hec1, 1:1000; anti-CENP-TW, 1:500, anti-Dsn1, 1:200; secondary antibodies, affinity-purified with horseradish peroxidase conjugate, 1:10000. After incubation with ECL western blotting system, images were acquired with the ChemiDoc™ MP Imaging System (BIO-RAD) in 16-bit TIFF format. Levels of images were adjusted using ImageJ software and then cropped and converted to 8-bit.

#### Immunofluorescence

Flp-In-T-Rex HeLa cells were plated on coverslips pre-coated with poly-L-lysine for 24 hr. Asynchronously growing cells were fixed using 4% paraformaldehyde. Cells were stained for CREST/anti-centromere antibodies (1:100), diluted in 2% BSA-PBS for 1.5 hr. Goat anti–human Alexa Fluor 647 was used as secondary antibody. DNA was stained with 0.5 μg/ml DAPI and coverslips were mounted with Mowiol mounting media. Preparations were examined under a microscope (MARIANAS, from 3i-Intelligent Imaging Innovations, Inc.) built around a stand (Axio Observer Z1; Zeiss) equipped with CSU-X1 confocal scanner unit (Yokogawa Electric Corporation) and a Plan-Apochromat 100x/1.4NA oil-immersion objective (Zeiss). Images were acquired as z sections at 0.27 μm. Images were converted into maximal intensity projections, exported and converted into 8-bit. Quantification of kinetochore signals was performed on unmodified 16-bit z series images using Imaris 7.3.4 32-bit software. After background subtraction, all signals were normalized to CREST. At least 728 kinetochores were analyzed per condition. Measurements were exported in Excel (Microsoft) and graphed with GraphPad Prism 6.0.

#### Protein Expression and Purification

A truncated version of the human MIS12C complex (MIS12C^Nano^) previously generated based on results of limited proteolysis experiments (Petrovic et al., 2014) was used as a starting point for structural and biochemical experiments. Constructs of MIS12 used in this study are described in Table S1B. *E.coli* BL21(DE3)-Codon-plus-RIL (or RIPL) cells containing the pST39 plasmid encoding a variant of the Mis12 complex under study, were grown in Terrific Broth (TB) at 37°C to an OD_600_ of 0.8. Protein expression was induced by the addition of 0.1 mM ITPG at 18°C and cells were incubated overnight for 16 hr. Cell pellets were resuspended in buffer A (20 mM Tris-HCl, pH 8, 300 mM NaCl, 10% (v/v) glycerol and 2 mM 2-mercaptoethanol supplemented with protease-inhibitor mix HP Plus and DNaseI, lysed by sonication and cleared by centrifugation. The cleared lysate was applied to 5 mL Ni-NTA-Fast Flow column pre-equilibrated in buffer A. The column was washed with 30 column volumes of buffer A containing 20 mM imidazole, and the bound protein was eluted with buffer A supplemented with 300 mM imidazole. The eluate was dialysed against ion exchange buffer A (20 mM Tris-Hcl, pH 8, 30 mM NaCl, 1 mM EDTA and 1 mM TCEP) and applied to a 6 mL Resource Q column pre-equilibrated in the same buffer. Elution of bound protein was achieved by a linear gradient from 30 mM to 300 mM NaCl in 20 column volumes. Relevant fractions were concentrated in 10 kDa molecular mass cut-off Amicon concentrators and applied to a Superdex 200 10/300 column equilibrated in size-exclusion chromatography buffer (20 mM Tris-HCl, pH 8, 150 mM NaCl and 1 mM TCEP). Size-exclusion chromatography was performed under isocratic conditions at a flow rate of 0.4 ml/min, and the relevant fractions were pooled, concentrated, flash-frozen in liquid nitrogen and stored at −80°C. Other Mis12 variants used in this study were purified using identical conditions. The expression of Head1 and Head2 constructs (MIS12:PMF1 and NSL1:DSN1 respectively, see Table S1B) was performed in *E.coli* BL21(DE3)-Codon-plus-RIL (or RIPL) cells. Protein expression was induced by the addition of 0.4 mM IPTG at 18°C and cells were incubated overnight for 16 hr. Cell pellets were resuspended in GST binding buffer (20 mM Tris-HCl, 300 mM NaCl, 10%(v/v) glycerol, 1 mM EDTA and 1 mM TCEP) supplemented with protease-inhibitor mix HP Plus and DNase I, lysed by sonication and clarified by centrifugation. The cleared lysate was incubated with Amintra Glutathione resin for two hours at 4°C. Following extensive washing (50-100 bead volume), the mixture was incubated with GST-3C protease (generated in house) overnight. The flow-through fraction was dialyzed against ion-exchange buffer A (see above), applied to a 6 mL Resource Q column (in case Head1) or 6 mL Resource S (in case of Head2), pre-equilibrated in the same buffer and the sample eluted using a linear gradient (from 30 to 300 mM NaCl for Head1 or 30 to 500 mM NaCl for Head2) over 20 column volumes. Relevant fractions were pooled, concentrated (10 kDa molecular mass cut-off Amicon concentrators) and applied to a Superdex 75 (10/300 or 16/600) column equilibrated in SEC buffer (20 mM Tris-HCl, pH 8, 150 mM NaCl and 1 mM TCEP). The sample was eluted under isocratic conditions, at a flow rate of 0.4 ml/min (for 10/300) or 1 ml/min (for 16/600), relevant fractions pooled, concentrated and flash-frozen. During purification of the Head2 construct, a stable proteolytic fragment generated by removal of residues near the N-terminal region of the DSN1 subunit was observed. The proteolytic fragment was separated from the full-length protein during the Resource S step, and was used in crystallization trials (vide infra).

In order to increase yield, MIS12C^Nano^ construct was also generated for the expression in insect cells. Expression and purification of the MIS12C^Nano^ complex was carried out in insect cells using a MultiBac system. Production of high-titer V_2_ virus was carried out separately for pFL-PMF1:MIS12 and pFL-DSN1-6xHis-NSL1 in Sf9 cells. Tnao38 insect cells (Hashimoto et al., 2012) were used for expression (96 hr, 27°C), after which the cells were centrifuged, washed once in PBS, re-centrifuged and frozen. Purification of MISC12^Nano^ was carried out in the same manner, as for the samples generated in *E. coli*.

CENP-C^1-71^ (Screpanti et al., 2011) was expressed in *E. coli* BL21(DE3)-Rosetta cells harboring the pGEX6P-2rbs-CENP-C^1-71^ plasmid. Cells were grown in TB to an OD_600_ of 0.8. Protein expression was induced by the addition of 0.2 mM ITPG at 18°C and cells were incubated overnight for 16 hr. Cell pellets were resuspended in GST binding buffer (20 mM Tris-HCl, 500 mM NaCl, 10%(v/v) glycerol, 1mM EDTA and 1 mM TCEP) supplemented with protease-inhibitor mix HP Plus and DNase I, lysed by sonication and clarified by centrifugation. The cleared lysate was applied to a 5 mL GST-Fast Flow column, pre-equilibrated in the GST binding buffer. Following extensive washing (50-100 column volume), GST-3C protease (generated in house) was added and the mixture incubated overnight. The flow-through fraction was concentrated (3 kDa molecular mass cut-off Amicon concentrators) and sample applied to a Superdex 75 (16/600) column, pre-equilibrated in CENP-C SEC buffer (20 mM Tris-HCl, pH 8, 300 mM NaCl and 1 mM TCEP). The sample was eluted under isocratic conditions, at a flow rate of 1 ml/min, relevant fractions pooled, concentrated and flash-frozen.

#### Crystallization

Prior to crystallization trials, purified MIS12C^Nano^:CENP-C^1-71^ complex was mixed with 1 mg/ml solution of α-Chymotrypsin at a ratio 1:1000 (w/w). Initial crystallization hits of MIS12C^Nano^:CENP-C^1-71^ were obtained in sitting drop crystallization experiments at ca. 10 mg/ml in a 96 well format using a Mosquito protein crystallization robot (TTP Labtech) at 4°C. Crystals grew as shower of thin plates, in range of conditions of various commercial screens including JCSG CoreI conditions B3 and D10, JCSG Core II conditions C10 and D100 and Procomplex condition D11 within 24-48 hr, reaching maximum size in 4-6 days. Crystals were further optimized in 96-well sitting drop iQ plates using a two-dimensional grid screen varying PEG6K (from 6%–12%) and pH (from 6-8). Crystals obtained in this way were also used as a source for seeds. In general, seeds were used in streak-seeding experiments employing a cat-whisker to optimize crystals in 24-well hanging-drop experiments. Streak-seeding from non-substituted protein was also used to facilitate growth of selenomethionine crystals. In general, MIS12C^Nano^:CENP-C^1-71^ complexes purified from *E. coli* and insect cells were used. For phasing, crystals were soaked overnight in mother liquor containing Ta_6_Br_12_ ranging in concentration from 0.5 mM to 2 mM. Crystals were cryo-cooled in a mother liquor solution containing 20%–25% (v/v) glycerol or 20% (v/v) ethylene glycol.

The MIS12C^ΔHead2^:CENP-C^1-71^ was crystallized at approximately 9.2 mg/ml in sitting drop crystallization experiments in a 96-well plate format. Initial crystals were obtained after 5 days at 4°C in JCSG Core I condition B10 (20% (w/v) PEG 3350, 0.2 M Potassium Sodium Tartrate). Crystals were cryo-cooled in a mother liquor solution containing 20% (v/v) glycerol.

We also obtained crystals of the isolated Head2 domain, generated by co-expression of N-terminal segments of DSN1 and NSL1 (Table S1B). Nonspecific proteolysis of the N-terminal DSN1 region (residues 68-106) occurred during purification. The Head2 proteolytic fragment was crystallized at approximately 15 mg/ml in sitting drop crystallization experiments in a 96-well plate format. Initial crystals were obtained at 20°C in JCSG Core III condition F10 (0.2 M Potassium Sodium Tartrate, 0.1 M Tri-Sodium Citrate, pH 5.6, and 2M Ammonium Sulfate). Crystals were cryo-cooled in a mother liquor solution containing 20% (v/v) glycerol.

#### Crystal Structure Determination

All data were collected at 100K using a Pilatus 6M detector either at the X10SA beamline at the SLS in Villigen, Switzerland, or at the P11 beamline of PETRA in Hamburg, Germany. All datasets were integrated and scaled using XDS and XSCALE and corrected for anisotropic diffraction using the UCLA Diffraction Anisotropy Server (except for the isolated Head2 domain data). The quality of the MIS12^Nano^:CENP-C^1-71^ crystals varied greatly in an unpredictable manner and necessitated screening of a large number of crystals. The best diffracting native crystals (3.5 Å) obtained by in situ proteolysis belonged to space group C2 with one molecule per asymmetric unit and were difficult to reproduce. Crystals obtained in conditions similar to the standard ones (see Crystallization), grew in a different space group (P2_1_) with two molecules per asymmetric unit and were soaked with a Tantalum bromide cluster. They diffracted to 5Å with a significant anomalous signal up to 6.5 Å. Phasing with PHENIX and SHARP located three binding sites for the Ta_6_Br_12_ cluster and allowed unambiguous positioning of alpha helices into the electron density. Molecular replacement with PHASER could successfully place the initial α-helical model into the native dataset in the C2 space group. Multi-crystal averaging of the native C2 dataset and another crystal in space group P1 (SeMet protein, but collected at 0.999 Å, that diffracted to 4.5 Å) with dmmulti (CCP4 package) greatly improved the density up to a point where refinement with PHENIX started to lower the free R factor. Separate masks were used for Head1, Head2 and the Stalk. Initially, the PHENIX option “autobuild from fragments” was used to improve the electron density. Later, secondary structure constraints and optimized weights were employed to prevent over-fitting. The sequence was assigned with the help of the structure of the *K. lactis* MIS12 complex (Dimitrova et al., 2016), which was fitted segment-wise to the human complex, accompanied by extensive analysis and comparison of secondary structure predictions (using PSIPRED) and complemented by cross-linking-mass spectrometry data. The anomalous signal of the P2_1_ SeMet crystal (collected at SeMet wavelength, diffracting to 6 Å) extended up to only ∼10A, and was therefore of limited use to the sequence assignment, but was used to confirm the positions of most of the selenomethionines. The CENP-C sequence was modeled into an F_obs_-F_calc_ electron density map after refinement of the protein was essentially complete and further lowered the free R value to a final 29.8% with acceptable Ramachandran geometry (91.8% favored, 6.6% allowed and 1.6% outliers).

The crystal structure of the MIS12C^ΔHead2^ was solved using molecular replacement with PHASER. It contained 2 molecules per asymmetric unit in a distinct P2_1_ crystal form. Head1 and the stalk were used as separate search models since no solution could be obtained with the model of full-length MIS12C after removal of Head2. Indeed, Head1 and the Stalk showed some hinge-bending motion of the two domains in comparison to the full-length structure in the C2 spacegroup. Rigid body refinement of the domains and separate helices lowered the free R factor. The slightly better resolution of the MIS12C^ΔHead2^ crystals compared to the full-length structure facilitated sequence assignment and further refinement using PHENIX. In spite of a high R factor, data up to 3.0 Å were initially included in refinement as they improved its convergence, whereas in the final refinement data to 3.25Å was used. The final model built on density for the MIS12C^Nano^ crystals contains residues 2-200 of MIS12, residues 31-203 of PMF1, residues 116-155, 159-193, 203-245, and 258-317 of DSN1, residues 32-204 of NSL1, and residues 6-22 and 28-48 of CENP-C.

The structure of the MIS12C^Head2^ was solved by molecular replacement, using the structure of the Head2 derived from the MIS12C^Nano^:CENP-C^1-71^ as a search model in Molrep (Collaborative Computational Project, Number 4, 1994). It contained 1 molecule per asymmetric unit in spacegroup P3_2_. The structure was rebuilt with Coot and refined with Refmac5, in iterative cycles until R-factors converged. The final refinement was done with PHENIX.

#### Fluorescence Polarization

Fluorescence polarization measurements were performed with a Safire 2 instrument (Tecan) at 30°C. The reaction volume was 20 μL and the fixed concentrations (20 nM) of 5-FAM labeled CENP-C^1-21^ peptide were mixed with increasing concentrations of the respective Mis12 variant (in the range of 1.28 pM-30 μM) in binding buffer (20 mM Tris-HCl, pH 8, 150 mM NaCl and 1 mM TCEP) in Corning 384 Well Low Volume Black Round Bottom Polystyrene NBS microplates. The reaction mixtures were allowed to equilibrate for approximately 15 min at room temperature. Fluorescein (5-FAM) was exited with polarized light at 470 nm, and the emitted light was detected at 525 nm through both horizontal and vertical polarizers. No change in the observed signal (or the underlying observed dissociation constant) was detected after one-hour incubation on ice. Polarization values are shown as mean ± standard error of the mean for three replicates and are plotted as a function of the logarithm of Mis12 concentration. Dissociation constant values (Kd) were obtained by fitting the fluorescence polarization data by non-linear least square method using the Origin software.

#### Protein Labeling

For site-specific protein labeling, Sortase mediated method was implemented. The C-terminal end of CENP-C^1-71^ was engineered to encode C-terminal sortase recognition motif (**LPETG**G) followed by a hexahistidine tag. The *S. aureus* SortaseA^Δ59^ expression plasmid was a kind gift of Hidde Ploegh. For labeling reaction, CENP-C^1-71^ was mixed at a concentration of 50 μM with 150 μM SortaseA and 1 mM synthetic peptide GGGK (labeled via lysine to 5-FAM) in reaction buffer (50 mM Tris-HCl, pH 7.5, 150 mM NaCl and 10 mM CaCl_2_) and incubated overnight at 4°C protected from light. Following overnight incubation, labeled CENP-C^1-71^ was separated from Sortase A and non-labeled CENP-C^1-71^ by passage through Nickel beads equilibrated in CENP-C SEC buffer (20 mM Tris-HCl, pH 8, 300 mM NaCl and 1 mM TCEP). The labeled CENP-C^1-71^ peptide was separated from excess synthetic peptide through repeated centrifugation steps with 3 kDa molecular mass cut-off Amicon concentrators, until the synthetic peptide in the flow-through was not detectable. The concentration of the labeled ^FAM^CENP-C^1-71^ fragment was estimated by measuring the absorbance for fluorescein at 495 nm.

#### Analytical Size-Exclusion Chromatography

Analytical size-exclusion chromatography was carried out on a Superdex 200 Increase 5/150 (ÄKTAmicro system) column. The samples were eluted under isocratic conditions at 4°C in SEC buffer (20 mM Tris-HCl, pH 8, 150 mM NaCl and 1 mM TCEP) at a flow rate of 0.2 ml/min. Elution of proteins was followed by monitoring wavelengths at 280 and 495 nm. Proteins were mixed at 5 μM in a total volume of 50 μl, incubated for 30 min on ice, spun for 15 min in a bench-top centrifuge before each chromatographic step. Relevant fractions (50 μl) were analyzed by SDS-PAGE. Chromatographic runs containing labeled protein fragments were analyzed by SDS-PAGE, and in-gel fluorescence was detected using a ChemiDoc™ MP Imaging System (BIO-RAD).

#### Chemical Cross-linking and Mass Spectrometry

Cross-linking of the 10-subunit KMN assembly complex with a CENP-C fragment was performed by mixing 45 μg of the complex (at 1 mg/ml) with 650 μM of an equimolar mixture of isotopically light (d0) and heavy (d12) labeled BS3 (bis-sulfosuccinimidylsuberate) for 30 min at 37°C. The reaction was quenched by adding a final concentration of 100 mM ammonium bicarbonate for 20 min at 37°C. Cross-linked proteins were enzymatically digested by trypsin or AspN and cross-linked peptides were identified by tandem mass-spectrometry (Herzog et al., 2012, Walzthoeni et al., 2012). Cross-linked proteins were denatured by adding 2 sample volumes of 8 M urea and reduced by incubating with 5 mM TCEP at 35°C for 15 min. Proteins were alkylated with 10 mM iodoacetamide for 35 min at room temperature in the dark. Samples were proteolytically digested using either trypsin or AspN. For the trypic digest, proteins were first incubated with lysyl endopeptidase (1/50, w/w) for 2 hr at 35°C followed by adding trypsin (1/50, w/w) overnight at a final concentration of 1 M urea. For the AspN digest, the protease was added twice, first at a final concentration of 1.6 M Urea (1/50, w/w) for 2 hr at 35°C and second in 0.5 mM Urea (1/50, w/w) overnight. Proteolysis was stopped by the addition of 1% (v/v) trifluoroacetic acid (TFA). Acidified peptides were purified by reversed-phase chromatography on C18 columns (Sep-Pak). Eluates were dried, reconstituted in 20 μl of mobile phase (water/acetonitrile/TFA, 75:25:0.1) and cross-linked peptides were enriched on a Superdex Peptide PC 3.2/30 column. Fractions of the cross-linked peptides were analyzed by liquid chromatography coupled to tandem mass spectrometry using a LTQ Orbitrap Elite (Thermo Scientific) instrument. The cross-link fragment ion spectra were searched and peptides identified by the open-source software *xQuest* (Walzthoeni et al., 2012). The results were filtered according to the following parameters: score < 0.85, MS1 tolerance window of −4 to 4 ppm and score ≥ 22 and manually validated. False positive rates calculated by *xProphet* (2) were 0.04 for inter-protein cross-links and < 0.01 for intra-protein cross-links.

### Quantification and Statistical Analysis

For polarization experiments, values are shown as mean ± standard error of the mean (described in Method Details). For kinetochore localization experiments, quantification and statistical analysis (mean ± SEM) are described in the figure legends.

### Data and Software Availability

The accession numbers for the MIS12^Δhead2^, Head2, and MIS12:CENP-C full-length structures reported in this paper are PDB: 5LSJ, 5LSI, and 5LSK, respectively.

## Author Contributions

A.P., J.K., I.R.V., and A.M. designed the experiments. A.P, J.K., Y.L., J.J., S.W., and P.S. created expression constructs and purified proteins. K.O. and S.v.G. carried out experiments with HeLa cells. P.R. and F.H. carried out mass-spec analysis of cross-linked proteins. A.P. performed the SEC and fluorescence polarization analyses. A.P., J.K., I.R.V., and Y.L. performed crystallization trials. A.P., J.K., and I.R.V. collected and processed diffraction data. I.R.V., with contributions of A.P., determined the structure and built the model. Y.D., S.J., and S.C.H. shared unpublished structural data of the fungal MIS12 complex that facilitated model building of parts of the human MIS12 complex. A.P. and Y.D. coordinated the collaboration of the Dortmund and Boston groups. J.K. performed fitting in electron microscopy maps. A.M. wrote the paper with input from A.P, J.K., and I.R.V. A.M. coordinated the working team.

## Figures and Tables

**Table 1:** X-Ray Data Collection and Refinement Statistics

	MIS12C^Nano^ Native	MIS12C^Nano^ SeMet (1)	MIS12C^Nano^ TaBr	MIS12C^Nano^ SeMet (2)	MIS12C^ΔHead2^	Head2
Space group (main use)	C2 (refinement)	P1 (multi crystal averaging)	P2_1_ (phasing)	P2_1_ (sequence assignment)	P2_1_ (refinement)	P3_2_21 (refinement)
Wavelength	0.97863	0.99999	1.25224	0.97930	0.97857	0.9793
Source	SLS	SLS	SLS	SLS	SLS	PETRA
Detector	Pilatus 6M	Pilatus 6M	Pilatus 6M	Pilatus 6M	Pilatus 6M	Pilatus 6M
Mol/AU	1	2	2	2	2	1
a, b, c (Å)	146.0, 112.7, 90.8	81.8, 92.9, 105.7	91.9, 107.8, 136.2	82.0, 93.2, 106.3	68.2, 156.4, 76.0	59.6, 59.6, 82.34
α, β, γ (°)	90, 114.18, 90	93.0, 92.6, 116.3	90, 102.5, 90	90, 90, 90	90, 102.82, 90	90, 90, 120
Resolution (Å)	19.7–3.5 (3.59–3.50)^a^	46.2–4.46 (4.58–4.46)	48.6–5.00 (5.13–5.00)	46.4–6.0 (6.16–6.00)	45.5–3.0 (3.08–3.00)	43.75–2.0 (2.05–2.00)
R_sym_	18.1 (195.6)	9.9 (184.8)	9.7 (93.4)	9.5 (188.2)	35.1 (102.6)	24.2 (135.5)
*I*/σ*I*	7.16 (1.05)	5.22 (0.70)	10.59 (2.51)	7.52 (0.82)	9.47 (3.89)	10.63 (2.12)
Completeness (%)	99.0 (99.1)	97.5 (84.5)	99.6 (99.2)	97.9 (99.0)	99.9 (100.0)	99.9 (99.4)
Redundancy	6.76 (6.67)	3.42 (2.58)	6.86 (6.99)	3.51 (3.70)	23.70 (24.82)	19.1 (18.1)

**Refinement**						

Resolution (Å)	19.74–3.5				20.0–3.25	43.75–2.00
No. reflections	14,170				24,286	11,252
R_work_/R_free_ (%)	24.44/29.77				24.63/29.71	21.58/24.58

**No. atoms**						

Protein/ligands	6,192				9,621	1,288
Water	6				0	53
Average B (Å^2^)	74.61				58.56	28.24

**Rmsd**						

Bond lengths (Å)	0.002				0.004	0.003
Bond angles (°)	0.630				0.815	0.516

a Values in parentheses are for highest resolution shell.

## Supplemental Information

Document S1. Table S1, Related to Figure 1

Table S2. Cross-linking Mass Spectrometry Analysis of the KMN Network, Simplified Table, Related to Figure 6

Table S3. Cross-linking Mass Spectrometry Analysis of the KMN Network, Raw Data, Related to Figure 6
