# Supplementary material for: Structure of the MIS12 Complex and Molecular Basis of Its Interaction with CENP-C at Human Kinetochores
Source: Cell. 2016 Nov 3;167(4):1028–1040.e15. doi: 10.1016/j.cell.2016.10.005 (PMC5101189; doi:10.1016/j.cell.2016.10.005)
Supplement: Document S1. Table S1, Related to Figure 1 [file mmc1.pdf]

Cell, Volume *167*

Supplemental Information

Structure of the MIS12 Complex and Molecular Basis of Its Interaction with CENP-C at Human Kinetochores

Arsen Petrovic, Jenny Keller, Yahui Liu, Katharina Overlack, Juliane John, Yoana N. Dimitrova, Simon Jenni, Suzan van Gerwen, Patricia Stege, Sabine Wohlgemuth, Pascaline Rombaut, Franz Herzog, Stephen C. Harrison, Ingrid R. Vetter, and Andrea Musacchio

Table S1A *Synonyms of outer kinetochore proteins discussed in this study*

Protein	Synonym 1	Synonym 2	Synonym 3	Synonym 4	Synonym 5
KNL1	CASC5	AF15q14	Blinkin	Spc105	SPC7
ZWINT	ZW10-interacting protein 1	Zwint-1			
NDC80	HEC1				
NUF2					
SPC24					
SPC25					
PMF1					
MIS12					
NSL1	C1orf48	DC31	DC8	MIS14	
DSN1	C20orf172	MIS13			

Table S1B *MIS12C constructs used in this study*

Construct	MIS12 boundaries	PMF1 boundaries	NSL1 boundaries	DSN1 boundaries
Mis12C^{Nano}	Mis12$^{1-205}$	Pmf1$^{31-205}$	Nsl1$^{1-206}$	Dsn1$^{68-356}$
Mis12C^{Head1}	Mis12$^{\Delta 2-90}$	Pmf1$^{\Delta 2-127}$		
Mis12C^{Head2}			Nsl1$^{29-99}$<br>Nsl1$^{2-99}$	Dsn1$^{68-200}$
Mis12C$^{\Delta Head1}$	Mis12$^{\Delta 2-90}$	Pmf1$^{\Delta 2-114}$	Nsl1$^{1-206}$	Dsn1$^{68-356}$
Mis12C$^{\Delta Head2}$	Mis12$^{1-205}$	Pmf1$^{31-205}$	Nsl1$^{\Delta 2-91}$	Dsn1$^{\Delta 2-185}$
Mis12C^{Loopless}	Mis12$^{1-205}$	Pmf1$^{31-205}$	Nsl1$^{1-206}$	Dsn1$^{68-356\Delta 100-109}$
